# Sc(III) Complexes of Pyclen Derivative Ligands as Probes for Hypoxia: Synthesis, Chemical Characterization, ^44^Sc‐Radiolabeling, and Preclinical Assessment

**DOI:** 10.1002/chem.202502763

**Published:** 2025-11-18

**Authors:** Tibor Csupász, Bayar Dahman, Tamás Gyula Gál, István Kapus, Zita Képes, Dániel Szücs, György Trencsényi, Imre Tóth, Anikó Fekete, Gyula Tircsó

**Affiliations:** ^1^ Department of Physical Chemistry Faculty of Science and Technology University of Debrecen Egyetem tér 1 Debrecen H‐4032 Hungary; ^2^ Doctoral School of Chemistry Faculty of Science and Technology University of Debrecen Egyetem tér 1 Debrecen H‐4032 Hungary; ^3^ Department of Chemistry Faculty of Science University of Zakho Zakho Kurdistan Region 42002 Iraq; ^4^ Department of Nuclear Medicine and Translational Imaging Institute of Medical Imaging Faculty of Medicine University of Debrecen Nagyerdei krt. 98. Debrecen H‐4032 Hungary

**Keywords:** detecting hypoxia, macrocyclic ligand complexes, nitroimidazoles, radioisotopes of Sc(III), physicochemical characterization of metal complexes

## Abstract

Rigid pyclen derivative ligands bearing hypoxia‐sensitive nitroimidazole (NI) pendants, PC2A‐Ph‐NI (hexadentate) and PC2AM‐NI (heptadentate), were synthesized, radiolabeled with ^44^Sc(III), and evaluated under preclinical circumstances. Physicochemical characterization was carried out using the structurally related model ligand, PC2AM^nBu^, by multinuclear NMR, UV‐Vis spectroscopy, and pH‐potentiometry. Sc(III) ion was found to form a stable complex with PC2AM^nBu^ (log *K*_ScL_ = 19.53(4); pSc = 19.32), in a reasonably fast reaction, with outstanding inertness (*t*_1/2_ = 529 hours in 1 M HClO_4_). The [Sc(PC2AM^nBu^)]^+^ complex can coordinate fluoride (log *K*_ScLF_ = 2.5(2)), and the ternary complex is remarkably inert against fluoride exchange. Based on these results, the PC2A‐mono(amide) platform seems to be a promising Sc(III)‐binder, paving the way for the labeling of NI‐containing ligands with ^44^Sc(III). Followed by successful radiolabeling with the positron‐emitting ^44^Sc isotope, labeling yields of 92% and over 99% were evidenced for [^44^Sc][Sc(PC2AM‐NI)]^+^ and [^44^Sc][Sc(PC2A‐Ph‐NI)]^+^; respectively, at 95 °C and 15 minutes; moreover, after purification, the radiochemical purity exceeded 99%. Both radiocomplexes remained stable in rat blood serum for at least 4 hours and exhibited high resistance toward transmetalation and transchelation processes. MicroPET imaging with preclinical models of B16‐F10 mouse melanoma was conducted to assess the tumor targeting capability and the in vivo biodistribution pattern of [^44^Sc][Sc(PC2AM‐NI)] and [^44^Sc][Sc(PC2A‐Ph‐NI)]^+^ radiopharmaceuticals. Given the higher tumor uptakes of [^44^Sc][Sc(PC2A‐Ph‐NI)]^+^ with decreasing off‐target activity and related better tumor/noise ratios, we may conclude that the diagnostic potential of the PC2A‐Ph‐NI derivative outperforms that of the [^44^Sc][Sc(PC2AM‐NI)]^+^.

## Graphical abstract

Hypoxia‐sensitive nitroimidazole‐functionalized pyclen derivatives were successfully synthesized and radiolabeled with ^44^Sc(III), affording stable and inert scandium complexes. Remarkable Sc(III)‐binding capabilities of the PC2A‐mono(amide) platform enabled excellent radiolabeling performance, with ≥92–99% yields and ≥99% radiochemical purity. MicroPET studies confirmed efficient tumor accumulation, with the PC2A‐Ph‐NI derivative exhibiting superior biodistribution and tumor‐to‐background ratios, highlighting its potential as a diagnostic radiopharmaceutical.

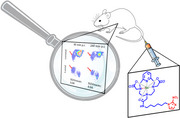

## 1 Introduction

Positron emission tomography (PET) is a sensitive, noninvasive, and functional imaging technique that allows for the quantitative in vivo assessment of the 3D distribution of radiopharmaceuticals with positron emission.^[^ ^1^ ^]^ Using PET in pathological processes could be detected even before morphological changes occur, at early disease stages.^[^ ^2^ ^]^ Within the radiopharmaceuticals, the vector molecule ensures the delivery of the radioactive component to lesions and that the emitted radiation accumulates at the desired target site, for example, tumoral cells.^[^ ^3^ ^]^

Permitting a simple radiolabeling procedure for different vector molecules, the chelation of radiometals is a favorable approach for routine clinical use.^[^ ^3^ ^]^ Due to its excellent nuclear properties,^[^ ^4^, ^5^, ^6^ ^]^ the positron‐emitting ^44^Sc isotope with a half‐life of 3.97 hours is a promising radiometal for PET imaging applications and, together with its isotopic counterpart ‐ the beta emitter ^47^Sc (half‐life: 3.35 d) is suitable for theragnostic applications as well.^[^ ^7^, ^8^ ^]^

Hypoxia plays a fundamental role in cancer development by inducing the formation of new capillaries from existing blood vessels that ensure oxygen and nutrient supply for tumor cell survival and proliferation tumor.^[^ ^9^ ^]^ Tumors with low oxygenation generally indicate a poorer prognosis due to increased aggressiveness, higher metastatic potential, and greater resistance to radio‐and chemotherapy.^[^ ^10^, ^11^ ^]^ Therefore, noninvasive assessment of tumor hypoxia by PET imaging is gaining increasing interest among clinicians. 2‐Nitroimidazole (NI) derivatives are excellent vector molecules for the visualization of hypoxic tumor regions due to their bioreductive trapping mechanism in low‐oxygenated tumor cells. Using radiolabeled 2‐NI compounds, tumor oxygenation levels can be determined by the PET technique.^[^ ^12^, ^13^ ^]^

**Figure 1:**
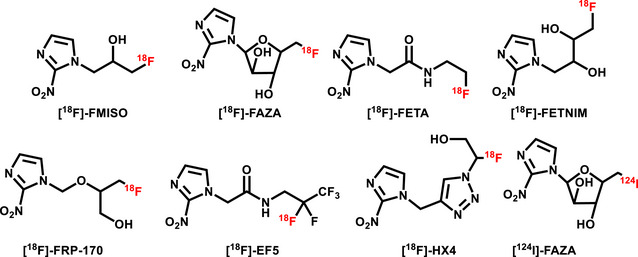
Some of the ^18^F‐ and ^124^I‐based probes proposed for hypoxia detection.

**Figure 2:**
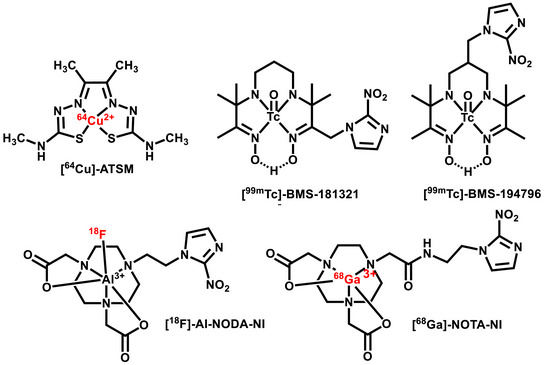
Formulae of some metallic radioisotope‐based probes for hypoxia detection.

Currently, [^1^ ^8^F]fluoromisonidazole ([^1^ ^8^F]FMISO) remains the most extensively utilized PET radiopharmaceutical for hypoxia imaging in humans.^[^ ^14^ ^]^ Nevertheless, its relatively slow uptake and clearance kinetics with related reduced contrast between hypoxic and normoxic tissues limit widespread application.^[^ ^12^, ^14^ ^]^ To address these shortcomings, alternative fluorine‐18‐labeled radiotracers have been developed. Possessing improved pharmacokinetic properties, one such radiopharmaceutical ‐ 1‐(5‐[^1^ ^8^F]fluoro‐5‐deoxy‐α‐D‐arabinofuranosyl)‐2‐NI ([^1^ ^8^F]FAZA) ‐ ensures enhanced tissue uptake and better hypoxia‐to‐normoxia contrast.^[^ ^12^ ^]^ Aside from [^18^F]FAZA, several other fluorinated NI derivatives are currently under investigation, including [^1^ ^8^F]fluoroetanidazole ([^1^ ^8^F]FETA), [^1^ ^8^F]fluoroerythronitromidazole ([^1^ ^8^F]FETNIM), 1‐(2‐[^1^ ^8^F]fluoro‐1‐[hydroxymethyl]ethoxy)methyl‐2‐NI ([^1^ ^8^F]RP‐170), 2‐nitroimidazol‐[^1^ ^8^F]pentafluoropropyl acetamide ([^1^ ^8^F]EF5), and [^1^ ^8^F]flortanidazole ([^1^ ^8^F]HX4) (Figure 1).^[^ ^15^, ^16^ ^]^ In addition to fluorine‐based tracers, metallic radioisotope‐labeled compounds have also been investigated as potential markers for hypoxia. Out of these, copper‐based agents, particularly the Cu‐diacetyl‐bis(*N*⁴‐methylthiosemicarbazone) (Cu(ATSM)) complex, were the earliest studied.^[^ ^17^, ^18^, ^19^, ^20^, ^21^, ^22^ ^]^ Despite initial promise, given the unclear mechanism behind their hypoxia selectivity, the use of these compounds remains limited. Furthermore, ⁶⁸Ga,^[^ ^23^, ^24^ ^]^^1^ ^24^/^1^ ^25^/^1^ ^3^ ^1^I,^[^ ^25^, ^26^, ^27^ ^]^ and ⁹⁹ᵐTc ^[^ ^28^ ^]^ radioisotopes, as well as the Al^18^F ^[^ ^29^ ^]^ labelling strategy, have also been used for radiolabelling in the development of hypoxia‐specific radiopharmaceuticals (Figure 2).

The positron‐emitting radionuclide ⁴⁴Sc has recently attracted considerable attention as a radiometal of high potential for PET imaging, primarily due to its favorable nuclear decay properties and suitable physical half‐life. ⁴⁴Sc exhibits a half‐life of 3.97 hours (recently refined to 4.04 hours), an average positron energy (E_β+_ average) of 632 KeV, and a positron emission intensity of 94.3%.^[^ ^8^, ^30^, ^31^ ^]^ Its intermediate half‐life enables delayed imaging protocols, enhancing lesion‐to‐background contrast and diagnostic accuracy, while maintaining logistical compatibility with routine radiopharmaceutical preparation and distribution workflows. In addition, the relatively low positron energy of ^44^Sc – consistent with the inverse relationship between positron energy and spatial resolution – results in improved reconstructed image quality and higher spatial resolution compared with other clinically employed PET isotopes, such as ^68^Ga.^[^ ^32^ ^]^ Taken together, these characteristics not only establish ⁴⁴Sc as an attractive candidate for PET applications but, in combination with its therapeutic counterpart ⁴⁷Sc, the ⁴⁴Sc/⁴⁷Sc theranostic pair offers a coherent platform for personalized radionuclide therapy as well as longitudinal monitoring of treatment efficacy.

**Figure 3:**
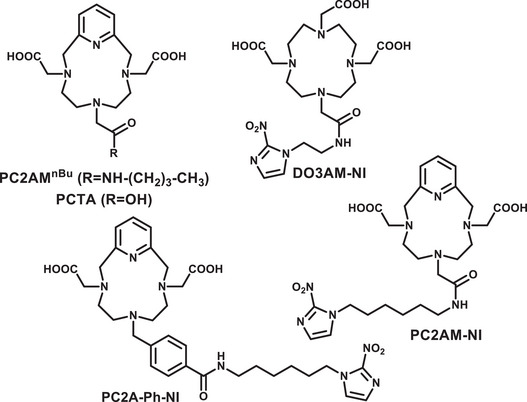
Formulae of the ligands studied (PC2AM^nBu^, PC2AM‐NI, and PC2A‐Ph‐NI) and used as comparative benchmarks (PCTA and DO3AM‐NI).

More recently, we have shown that a ^44^Sc complex formed by a DO3A‐mono(amide) ligand bearing an NI moieties as a hypoxia probe (DO3AM‐NI),^[^ ^33^ ^]^ thereby enabling the mapping of hypoxic tumor regions with PET imaging. In this study, a DOTA‐derived chelator was used for complexing Sc(III) ion; however, 1,4,7‐triazacyclononane (TACN) and AAZTA (6‐amino‐6‐methylperhydro‐1,4‐diazepinetetraacetic acid) derivatives are also commonly employed for such purposes.^[^ ^34^, ^35^, ^36^, ^37^, ^38^, ^39^, ^40^ ^]^ To the best of our knowledge, chelators based on the pyclen platform have not yet been employed for Sc(III) complexation. However, literature reports indicate that rigidification of the 1,4,7,10‐tetraazacyclododecane macrocyclic framework has a beneficial effect on the apparent stability and kinetic inertness of the resulting complexes. Therefore, we selected PCTA (Figure 3) and its derivatives, which are widely used for the complexation of Ln(III) ions‐notably, the ligand of the newest Gd(III)‐based MRI contrast agent, Gadopiclenol (marketed as Elucirem by Guerbet and Vueway by Bracco), is a pyclen derivative) ^[^ ^41^ ^]^ ‐ as well as for Mn(II) ion ^[^ ^42^, ^43^ ^]^ and, more recently, Ga(III) ions,^[^ ^44^ ^]^ the later as a possible fluoride carrier. To address this issue, we herein determined the stability and the acid‐assisted dissociation of the Sc(III) complexes formed under acidic conditions with PCTA and its mono(amide) derivative, PC2AM^nBu^, employed as a model compound for physicochemical characterization (synthesized as a model compound to PC2AM‐NI). In addition, we also aimed to study the interaction between the [Sc(PC2AM^nBu^)]⁺ complex and fluoride ions, as well as the kinetics of fluoride exchange in the resulting ternary complex ([Sc(PC2AM^nBu^)(F)] using ^1^⁹F‐NMR spectroscopy. Furthermore, to construct hypoxia‐targeting PET probes, both previously synthesized PC2A‐Ph‐NI (hexadentate) and PC2AM‐NI (heptadentate) ligands containing an NI moiety were labeled with the [⁴⁴Sc]Sc(III) radioisotope, and then the hypoxia sensitivity of the resulting radiotracers was tested in vivo using B16‐F10 melanoma tumor‐bearing C57BL/6 mice and microPET imaging. These ligands were originally designed for the complexation of Mn(II) ions, as they are expected to form either mono‐aqua complexes (applicable for both PET and MRI applications) or complexes without a coordinated water molecule (thus intended solely for PET applications). However, our recent study has shown that pyclen derivative ligands (OPC2A = 6‐oxa‐3,9,15‐triazabicyclo(9.3.1)pentadeca‐1(15),11,13‐triene‐3,9‐diacetic acid) may also be suitable for the complexation of Sc(III) ions.^[^ ^45^ ^]^

## 2 Results and Discussion

### 2.1 Synthesis of PC2AM^nBu^ Ligand

**Figure 4:**
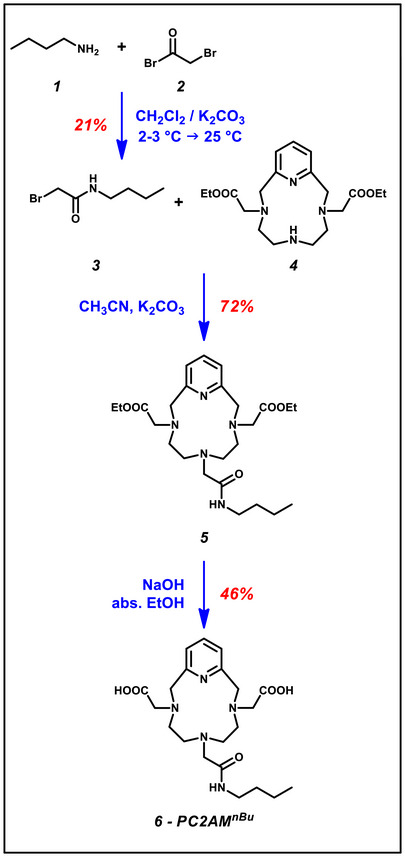
The synthesis of PC2AM^nBu^.

The PC2AM^nBu^ model compound used in equilibrium and kinetic studies was obtained by alkylating 3,9‐PC2A^OEt^ (4) with 2‐bromo‐N‐butylacetamide (3) prepared by the acylation reaction of butylamine (1) with bromoacetyl bromide (2) in a two‐step reaction. First, the secondary amine group of the 3,9‐PC2A^OEt^ (4) compound was reacted with 2‐bromo‐N‐butylacetamide (3), and the reaction product was purified from by‐products using preparative HPLC. Then, the ethyl protecting groups of PC2A^OEt^AM^nBu^ (5) were removed by saponification with NaOH to obtain PC2AM^nBu^ (6), the ligand used in the physicochemical studies (Figure 4).

### 2.2 Equilibrium Studies

The equilibrium study of the PC2AM^nBu^ ligand has been started by determining the protonation constants in 0.15 M and 1.00 M NaCl ionic strength. (As the stability of the Sc(III) complex was determined in 1.00 M ionic strength, we had to perform the pH‐potentiometric titration of ligands using 1.00 M ionic strengths also.) The results of pH‐potentiometric measurement are shown in Table 1, where the corresponding data for 3,9‐PC2A,^[^ ^42^ ^]^ PCTA ^[^ ^46^ ^]^ and PC2AM‐NI ligands determined under identical conditions are also presented. Knowledge of the protonation constants defined by Eqn. 1 and 2 are essential for the calculation of the stability constants of the Sc(III) complexes:

**Table 1:** Protonation constants of PC2AM^nBu^ along with the corresponding data of PC2AM‐NI, 3,9‐PC2A ^[^ ^42^ ^]^ and PCTA ^[^ ^46^ ^]^ ligands (*T* = 25 °C).

	PC2AM^nBu^		PC2AM‐NI	3,9‐PC2A	PCTA	
*I*	0.15 M NaCl	1.00 M NaCl	0.15 M NaCl	0.15 M NaCl	0.15 M NaCl ^[^ ^46^ ^]^	1.00 M NaCl
log *K*_1_^H^	**8.79(1)**	**8.13(2)**	**8.60(3)**	12.25	9.97	**9.22(6)**
log *K*_2_^H^	**7.59(1)**	**7.59(1)**	**7.39(3)**	5.97	6.73	**7.30(8)**
log *K*_3_^H^	**2.31(2)**	**2.52(1)**	**2.28(3)**	3.47	3.22	**4.09 (9)**
log *K*_4_^H^	**1.28(3)**	‐	**1.58(3)**	1.99	1.40	**1.42(8)**
log *β*_2_^H^	***16.38***	***15.72***	***15.99***	*18.22*	*16.70*	***16.52***

(1) $$\mathrm{PC}2{\mathrm{AM}}^{{\mathrm{nBu}}^{2-}}+{\mathrm{nH}}^{+}⇌H_{n}{\left(\mathrm{PC}2{\mathrm{AM}}^{\mathrm{nBu}}\right)}^{-2+n}$$

(2) $$\beta_{n}=\frac{\left[H_{n}{\left(\text{PC}2AM^{\text{nBu}}\right)}^{-2+n}\right]}{\left[\text{PC}2A{M^{\text{nBu}}}^{2-}\right]{\left[H^{+}\right]}^{n}}$$

Table 1 shows that the nitrogen donors of the 3,9‐PC2A ligand have the highest basicity (log *β*_2_^H^), with the largest contribution from the secondary amine group in the macrocycle backbone. For the amide derivatives, the electron‐withdrawing groups attached to the N‐atom at the 6^th^ position reduce the basicity of the amine group; therefore, the first protonation constants are more than 3 orders of magnitude lower. Compared to the first protonation constants of the PCTA ligand, the corresponding values of the PC2A‐mono(amide) (PC2AM) derivatives are lower by one order of magnitude, which can be interpreted as a reduction in basicity due to the substitution of an acetate group by an amide pendant. The second protonation of the pyclen‐type ligands occurs on one of the *cis*‐nitrogen atoms of the macrocycle, while the first proton migrates from the *trans*‐nitrogen atom to the second *cis*‐nitrogen atom simultaneously, resulting in a “better” charge distribution, that is, in smaller repulsion between the two protons. In fact, the second protonation constants characterizing this process are larger for PC2AM derivatives than those measured for PCTA and 3,9‐PC2A ligands. It is also clear that there is no significant difference in the basicity of PC2AM^nBu^ and PC2AM‐NI; this finding validates our selection of using PC2AM^nBu^ as a model compound of PC2AM‐NI. However, the lower log *β*_2_^H^ and the lower overall basicity (log *β*_4_^H^) of the PC2AM derivatives predict the lower stability of their complexes with metal ions as compared to PCTA.

**Figure 5:**
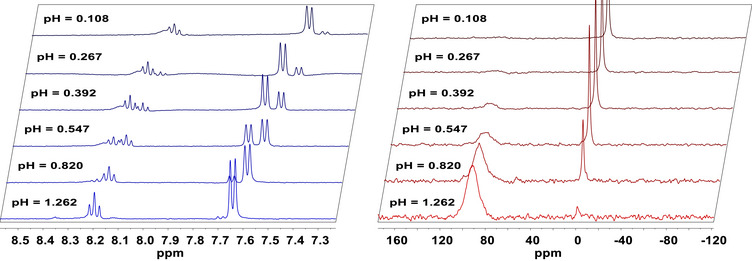
The aromatic region of 400 MHz ^1^H NMR (left) and 97 MHz ^45^Sc NMR (right) spectra of Sc(III) ‐ PC2AM^nBu^ ^2−^ ‐ H^+^ system at different ‐log[H^+^] values (H_2_O with 10% D_2_O, c_Sc _= 3.00 mM, c_ligand_ = 3.06 mM, 1.0 M NaCl/HCl, 298 K).

The stability constant of the complex of PC2AM^nBu^ ligand formed with Sc(III) ion was studied using “out‐of‐cell” (batch) samples using ^45^Sc NMR spectroscopy in strongly acidic media. Typical ^45^Sc NMR spectra are shown in Figure 5 along with the corresponding ^1^H NMR spectra of the aromatic region of the ligand.

The stability constant was defined as follows:

(3) $${\mathrm{Sc}}^{3+}+\mathrm{PC}2{\mathrm{AM}}^{{\mathrm{nBu}}^{2-}}⇌{\left[\mathrm{Sc}\left(\mathrm{PC}2{\mathrm{AM}}^{\mathrm{nBu}}\right)\right]}^{+}$$

(4)

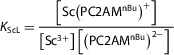




**Figure 6:**
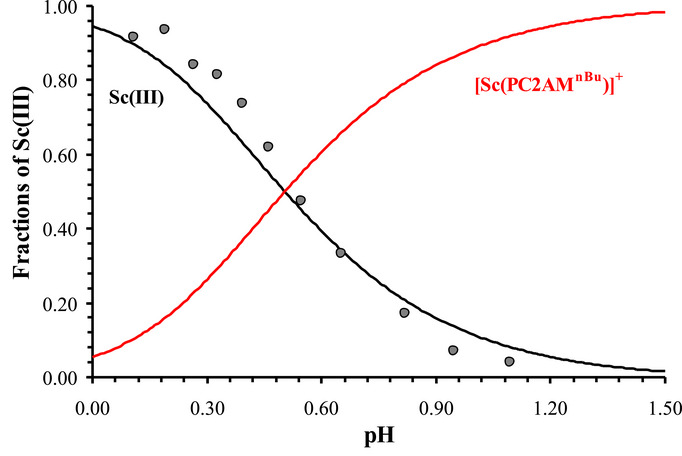
Calculated species distribution curves for the Sc^3+^ ‐ PC2AM^nBu^ ^2−^ ‐ H^+^ system as a function of ‐log[H^+^] values coplotted with the measured free Sc(III) concentration from ^45^Sc NMR (● symbols) (c_Sc(III)_ = 3.00 mM, c_ligand_ = 3.06 mM, 1.0 M NaCl/HCl, 298 K).

Using the ^45^Sc NMR signal intensities of free Sc^3+^ measured in the equilibrated samples, the stability constant, *K*_ScL_, was calculated by fitting the areas with the PSEQUAD program ^[^ ^47^ ^]^ (Figure 6). The best fitting was observed by considering the simplest model including only [Sc(PC2AM^nBu^)]^+^ species; that is, the formation of protonated [Sc(*H*PC2AM^nBu^)]^2+^ cannot be confirmed.

The stability constant of the [Sc(PCTA)] complex was determined in a similar way (Figure S13). The results were summarized in Table 2, including the data of [Sc(DO3AM‐NI)], determined in our previous study.^[^ ^33^ ^]^

**Table 2:** The stability constants and *p*Sc values of [Sc(PC2AM^nBu^)]^+^, [Sc(PCTA)] and [Sc(DO3AM‐NI)]^[^ ^33^ ^]^ complexes (*T* = 25 °C).

	PC2AM^nBu^	PCTA	DO3AM‐NI^[^ ^33^ ^]^
*I*	1.00 M NaCl	1.00 M NaCl	0.15 M NaCl
log *β*_2_^H^	15.72	16.52	17.38
log *K*_ScL_	19.53(4)	22.41(9)	22.36
*p*Sc^[^ ^a^ ^]^	19.32	21.28	20.74
A_L/M_^[^ ^b^ ^]^	5.17	7.14	6.57

^[a]^ *p*Sc = ‐ log [Sc(III)]_free_ (c_Sc(III)_ = 1x10^−8^ M; c_lig_ = 1x10^−7^ M at pH = 7.4) calculated by considering the hydrolytic equilibria of Sc(III);

^[b]^ A_L/M_ was calculated as suggested by M. Meyer^[^ ^48^ ^]^;

Among the complexes shown in Table 2, the [Sc(DO3AM‐NI)] complex has the highest stability, but its stability constant is lower than the corresponding values of [Sc(DOTA)]^−^ (log *K*_ScL_  =  30.79) and [Sc(DTPA)]^2−^ (log *K*_ScL_  =  27.43). ^[^ ^49^ ^]^ As evidenced for DO3AM‐mono(amide) the replacement of acetate side chains with amide groups results in a decrease in stability constant (and conditional stability); therefore, we can see an order of magnitude difference between the stabilities of [Sc(PCTA)] and [Sc(PC2AM^nBu^)]^+^. Although these *K*_ScL_ constants and *p*Sc values are somewhat lower than those of the complexes formed with traditional DOTA and DTPA ligands, these chelators remain remarkably good Sc(III)‐binding chelators. However, comparing pM values can be problematic in systems involving easily hydrolyzing metal ions under very diluted conditions (as these values do not account for hydrolyzed forms or processes that may compete with complex formation in very dilute solutions). Therefore, following the proposal of M. Meyer and colleagues,^[^ ^48^ ^]^ A_L/M_ values (relative affinity A_L/M_ = −log(∑[Sc(III)]_unbound_/[Sc(III)]_total_) = −log{([Sc(III)]_total_−∑[Sc(III)]_bound_)/[Sc(III)]_total_} where [Sc(III)]_total_ = 10 nM and [L]_tot_ = 100 nM at pH  =  7.4 whereas Figure S14 in the ESI displays its dependence on pH in a wide pH range), introduced as a universal tool for the reliable assessment and comparison of the complexing power of any ligand, were also calculated and presented in Table 2. For the A_L/M_ values, 1 corresponds to 90%, 2 corresponds to 99%, 3 corresponds to 99.9% etc. for complex formation. By examining the A_L/M_ data in Table 2, one can conclude that the [Sc(PC2AM^nBu^)]^+^ complex possesses lower A_L/M_ value than [Sc(PCTA)], which displays a higher A_L/M_ value than that of the [Sc(DO3AM‐NI)]. The A_L/M_ data of the [Sc(PC2AM^nBu^)]^+^ complex only exceeds that of the [Sc(DO3AM‐NI)] complex in the pH < 3.0 range, which can be advantageous for labeling at low pH. Furthermore, based on the data in Table 2, it can be concluded that for these systems there is no discrepancy between the trends observed in the pSc and A_L/M_ values.

In order to explore further the acid‐base properties of [Sc(PC2AM^nBu^)]^+^, pH‐potentiometric titration was carried out on a pre‐equilibrated sample. The sample with Sc(III): ligand  =  1:1 was prepared (c_Sc _ =  c_lig_ = 3 mM) one day before the titration by mixing the calculated volume of *H_2_* PC2AM^nBu^ and ScCl_3_ stock solutions (without the addition of any acid). The “aged” sample was acidified right before the titration by adding a known amount of acid to set the pH acidic (‐log[H^+^]_starting_  =  1.52) and titrated with standardized NaOH solution. The titration curve obtained displayed a typical “strong acid–strong base” titration curve, indicating the absence of mixed hydroxo species, [Sc(PC2AM^nBu^)(OH)], in the pH range studied (1.52–12.00). Our attempts to detect [Sc(*H*PC2AM^nBu^)]^2+^ from the same data set have also failed.

**Figure 7:**
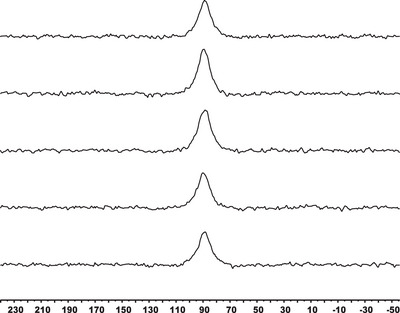
97.20 MHz ^45^Sc‐NMR spectra of [Sc(PC2AM^nBu^)]^+^ at different pH (at 3.12, 5.50, 7.00, 8.00, and 9.00 going from top to bottom; c_sc(III)_ = c_ligand_ = 3 mM, 0.15 M NaCl, 298 K, H_2_O with 10% D_2_O).

One could be surprised that the [Sc(PC2AM^nBu^)]^+^ complex (supposed to be 7 coordinated by the heptadentate (PC2AM^nBu^ and PC2AM‐NI) ligands, considering the weekly coordinating bulky amide group) does not contain a water molecule coordinated to Sc(III) on which deprotonation could occur, although Sc(aq)^3+^ is known as 8 coordinated.^[^ ^50^ ^]^ To confirm the absence of the mixed hydroxo species, we also performed ^45^Sc NMR measurements. In accordance with the pH‐potentiometry, only one ^45^Sc‐NMR signal contributed to the [Sc(PC2AM^nBu^)]^+^ complex, which was detected at 92 ppm in the pH range of 3–9; that is, ^45^Sc NMR spectra showed clearly that the formation of [ScL(OH)] was negligible until pH  =  9 (Figure 7). No change of ^1^H NMR spectra of the same samples was found, supporting the absence of mixed hydroxo complex in the system (Figure S15).

**Figure 8:**
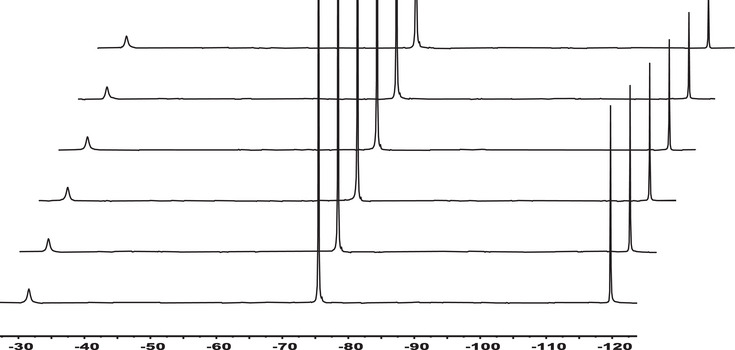
376.4 MHz ^19^F NMR spectra of 5 mM [Sc(PC2AM^nBu^)(F)] (pH kept at pH  =  5.2, I  =  0.15 M NaCl, 25 °C, H_2_O with 10% D_2_O) at different concentrations of added NaF (c_NaF_  =  10, 9, 8, 7, 6, and 5 mM from the bottom to the top). Chemical shift values: ‐31 ppm ([Sc(PC2AM^nBu^)(F)]); ‐120 ppm (free F^−^); ‐75 ppm (TFA impurity).

Sc(III) is a typical “hard” cation, forming very stable complexes with fluoride anion.^[^ ^51^, ^52^ ^]^ Although the [Sc(PC2AM^nBu^)]^+^ cation seems to be “saturated”, that is, it does not form a mixed hydroxo complex as it lacks coordinated water molecule, we have explored its interaction with fluoride ion using ^19^F NMR spectroscopy (Figure 8). As can be seen in the figure, a new signal appears at ‐31 ppm during the titration of a 5 mM solution of [Sc(PC2AM^nBu^)]^+^ at pH  =  5.2. The formation of the ternary [Sc(PC2AM^nBu^)(F)] complex can be followed by ^1^H NMR, as two sets of signals (Δδ ∼ 20 Hz) indicate a slow exchange regime on the ^1^H NMR time scale (Figure S16). Measuring the intensities of the free and bound fluoride signals, the stability constant (Eqn. 6) of the [Sc(PC2AM^nBu^)(F)] complex (Eqn. 5) can be calculated (Table S1).

(5) $${\left[\mathrm{Sc}\left(\mathrm{PC}2{\mathrm{AM}}^{\mathrm{nBu}}\right)\right]}^{+}+F^{-}⇌\left[\mathrm{Sc}\left(\mathrm{PC}2{\mathrm{AM}}^{\mathrm{nBu}}\right)\left(F\right)\right]$$

(6) $$K_{\mathrm{ScLF}\;}=\frac{\left[\mathrm{Sc}\left(\mathrm{PC}2AM^{\mathrm{nBu}}\right)\left(F\right)\right]}{\left[\mathrm{Sc}{\left(\mathrm{PC}2AM^{\mathrm{nBu}}\right)}^{+}\right]\left[F^{-}\right]}$$

The log *K*_ScLF_  =  2.5(2) indicates substantially strong interaction between the Sc(III) macrocyclic complex cation and the fluoride anion, and it is likely based on the replacement of one carboxylate (or amide) group of the ligand with fluoride. The ^45^Sc NMR chemical shift of the reasonably (ca. 100 Hz) broad signals of the parent and mixed fluoro complexes are 90±10 ppm and 65±15 ppm, respectively (Figure S17). Kelderman et al. found a correlation between the ^45^Sc NMR shift and the coordination number of the Sc(III) in different complexes formed by TACN (TACN = 1,4,7‐triazacyclononane) derivatives in methanol solvent.^[^ ^53^ ^]^ Based on Kelderman's classification, both complexes can be considered as hepta‐coordinate species.

Based on physicochemical studies demonstrating the ability of the [Sc(PC2AM^nBu^)]^+^ complex to bind fluoride, we decided to investigate the formation of the corresponding radiofluorinated ternary complex. The experimental conditions for this study are provided in the Supporting Information. In agreement with its relatively low stability, the [Sc(PC2AM^nBu^)]^+^ complex could be labeled with ^18^F, yielding a 56% radiochemical conversion. Although the given labeling yield requires further optimization, the present result provides a solid foundation to speculate on the suitability of such a platform as a fluoride carrier in radiochemical applications.

### 2.3 Kinetic Studies

#### 2.3.1 The time course of complex formation of [Sc(PC2AM^nBu^)]^+^ and [Sc(PCTA)] complexes

The time course of complex formation of PC2AM^nBu^ and PCTA ligands with Sc(III) ion was investigated by ^45^Sc‐NMR under acidic conditions (c_Sc_  =  c_ligand_ = 10 mM and 9.5 mM, respectively, ‐log[H^+^] = 1.28). The decrease of the narrow signal of the free Sc(III) ion was monitored as a function of time. Meanwhile, the signal of the complexes gradually increased, while the areas of the (broader) signals corresponding to the [Sc(PC2AM^nBu^)]^+^ (δ = 91 ppm) and [Sc(PCTA)] complexes (δ = 92 ppm) were also followed. The kinetic measurements of formation could be seen in Figures S18 and S19. The complexation for the [Sc(PCTA)] chelate at pH  =  1.28 and 25 °C took about 300 minutes to reach the equilibrium, whereas for the [Sc(PC2AM^nBu^)]^+^ complex it took a bit longer time under these conditions. (In order to confirm that the samples reached the equilibrium, we have repeated the ^45^Sc NMR study for both samples after a few days when the signal of the free Sc(III) was absent, and only the signals of the complexes were detected.) The detailed study of formation kinetics was not our aim in this project. One can expect that the reaction follows the well‐known mechanisms of M(III) ‐ macrocyclic APC systems ^[^ ^54^ ^]^: formation of a protonated intermediate in fast pre‐equilibrium followed by a slow, base‐catalyzed deprotonation/structural rearrangement as a rate‐determining step. The intermediate is named also as “out‐of‐cage complex.”^[^ ^55^ ^]^ Our experimental curves are likely in accordance with this mechanism. Fitting the measured data for the free Sc(III) signals by a monoexponential function gives the rate constant *k*_obs_ = (1.04 ± 0 .05) x 10^−4^ s^−1^ for the [Sc(PC2AM^nBu^)]^+^ and *k*_obs_ = (2.51 ± 0.12) x 10^−4^ s^−1^ for the [Sc(PCTA)] complex. These conditional constants can be converted into half‐lives of the reactions using *t*_1/2 _ =  ln2 / *k*_obs_ expression, resulting in 110‐minute and 46‐minute half‐lives, respectively. The relatively fast complex formation at the given acidic condition is very encouraging for the Sc(III) labeling experiments.

#### 2.3.2 Decomplexation of [Sc(PC2AM^nBu^)]^+^ and [Sc(PCTA)] complexes

**Figure 9:**
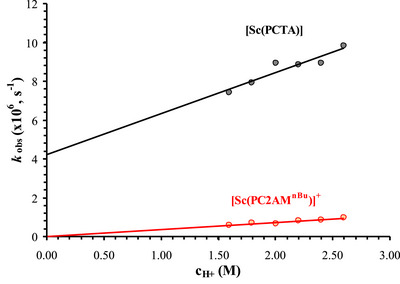
The pseudo‐first‐order rate constants observed for the dissociation of [Sc(PC2AM^nBu^)]^+^ (red symbols) (c_complex_  =  0.32 mM) and [Sc(PCTA)] (blue symbols) (c_complex_  =  0.30 mM) as a function of acid concentration (25 °C, *I*  =  2.60 M).

The inertness of metal complexes is a basic requirement in all relevant modalities of medical imaging. Complexes of macrocyclic ligands are often more inert compared to open‐chain analogs. In order to assess the behavior of our complexes, the decomplexation of [Sc(PC2AM^nBu^)]^+^ and [Sc(PCTA)] was studied in a strongly acidic medium (c_H__+_ = 1.6 ‐ 2.6 M HClO_4_) by using UV spectroscopy. The UV‐Vis spectra of the Sc(III) complexes and those of the free ligands differ significantly; the reaction has been followed at 280 nm (Figures S20 and S21). The primary Abs. versus time curves have been fitted by monoexponential function returning *k_obs_* (s^−1^) values (Figures S22 and S23). The *k*_obs_ versus c_H_^+^ graphs are shown in Figure 9.

The plots of the rate constants as a function of acid concentration yielded linear relationships for both complexes *(k*_obs _ =  *k*_0_ + *k*_1_[H^+^]). The slopes of these lines correspond to the rate coefficients characteristic for acid‐assisted dissociation (*k*_1_), whereas the intercepts characterize the acid‐independent part, often referred to as a rate of spontaneous dissociation. It is worth noting, however, that in such a strongly acidic medium, this may indicate that the observed process does not correspond to the spontaneous dissociation of the parent complex but rather to that of a protonated complex present in high concentration in solution. As can be seen in Figure 9, the two complexes possess remarkably different inertness; moreover, a notable y‐intercept is also observed for the [Sc(PCTA)] complex. This most likely indicates that the present complex has a strong tendency to form a protonated species in a strongly acidic solution, from which both the spontaneous (*k*_0_) and the acid‐assisted dissociation processes (*k*_1_) could be characterized. This, in turn, implies that the complex exhibits lower inertness. The rate coefficients obtained during the fitting are summarized in Table 3, along with the half‐lives calculated from the rate coefficients by using 1 M acid concentration (*t*_½_ = ln 2 / *k*_obs_).

**Table 3:** The rate constants and calculated half‐lives characterizing the dissociation of [Sc(PC2AM^nBu^)]^+^ and [Sc(PCTA)] complexes compared to that of [Sc(DOTA)]^[^ ^49^ ^]^ (25 °C, *I*  =  2.6 M NaCl).

	PC2AM^nBu^	PCTA	DOTA
*k*_0_ (s^−1^)	**0**	**(4.21 ± 0.81) x 10^−6^**	6.0 x 10^−6^
*k*_1_ (M^−1^s^−1^)	**(3.64 ± 0.08) x 10^−7^**	**(2.11 ± 0.38) x 10^−6^**	4.3 x 10^−6^
*t*_1/2_ (h) (1 M H^+^)	**529**	**30.5**	18.7

The rate of acid‐assisted dissociation of the [Sc(PCTA)] complex is in the same order of magnitude as that of the [Sc(DOTA)]^−^ complex,^[^ ^49^ ^]^ whereas the [Sc(PC2AM^nBu^)]^+^ monoamide derivative dissociates more than an order of magnitude slower under highly acidic conditions. The calculated half‐lives also indicate that the presence of the amide group in the ligand significantly improves the inertness of the complex. Altogether both [Sc(PC2AM^nBu^)]^+^ and [Sc(PCTA)] complexes are very inert, the former showing outstanding inertness required for the in vivo studies.

#### 2.3.3 Kinetics of fluoride exchange reaction

Considering ^18^F‐labeling and PET application of the [Sc(L)(F)]‐type mixed complexes, one has to check the inertness of the mixed complex against F‐exchange:

(7) $$\left[\mathrm{Sc}\left(\mathrm{PC}2{\mathrm{AM}}^{\mathrm{nBu}}\right)F\right]+*F^{-}⇌\left[\mathrm{Sc}\left(\mathrm{PC}2{\mathrm{AM}}^{\mathrm{nBu}}\right)*F\right]+F^{-}$$

where * is used to indicate typographically the exchanging anion.

The ^19^F NMR signal of the mixed complex is somewhat broadened (*ν*_½_ = 150 Hz) due to the relaxation effect of the quadrupole ^45^Sc (I  =  7/2) nuclei, while the free fluoride is narrow (*ν*_½_ = 4.5 Hz), indicating the absence of chemical exchange on the *T*_2_‐time scale (Figure S24). However, the selective magnetization transfer experiment shows chemical exchange between [Sc(PC2AM^nBu^)(F)] (site A) and free F^−^ (site B) with *k*_obs_^AB^ value of 0.6 s^−1^ (14% uncertainty), indicating remarkable inertness of the monodentate fluoride (Figure S25). Very recently, 3–4 orders of magnitude larger fluoride exchange rate constants have been published for two Y(III) complexes formed with open‐chain ligands, [Y(EGTA)(F)]^2−^ and [Y(OBETA)(H_2_O)(F)]^2−^.^[^ ^56^ ^]^ In the case of macrocyclic [Ln(DOTA‐tetramide)(F)]^2+^ systems (Ln  =  Yb, Eu), the rate constants are *k*_obs_  =  61 s^−1^ and 0.41 s^−1^, respectively, for the amide derivative possessing aromatic substituents, which are known to slow the rate of fluoride exchange as compared to that of a complex of simple tetra‐*N‐*methyl‐amides (DTMA) (*k*_obs_  =  216 s^−1^ for [Yb(DTMA)(F)]^2+^). ^[^ ^57^ ^]^ At the same time, the *k*_obs_ value obtained for the [Sc(OPC2A)(F)] complex (*k*_obs_  =  16.5 s^−1^) is lower by an order of magnitude than that of [Yb(DTMA)(F)]^2+ [^ ^45^ ^]^ which is likely to be rationalized by the more rigid environment around the M(III)‐F entity. However, the fluoride exchange rate of open‐chain [Al(EDTA)(F)]^2−^ much smaller (no magnetization effect was observed in the MT experiment for τ values up to 3,5 – 4 seconds, allowing for estimation of *k*_obs_ to be greater than or equal to 0.2 s^−1^),^[^ ^58^ ^]^ reflecting the role of the individual metal center, and preventing us from further speculation to find a simple structure‐inertness correlation.

### 2.4 Radiochemical studies of [^44^Sc][Sc(PC2AM‐NI)]^+^ and [^44^Sc][Sc(PC2A‐Ph‐NI)]^+^

**Figure 10:**
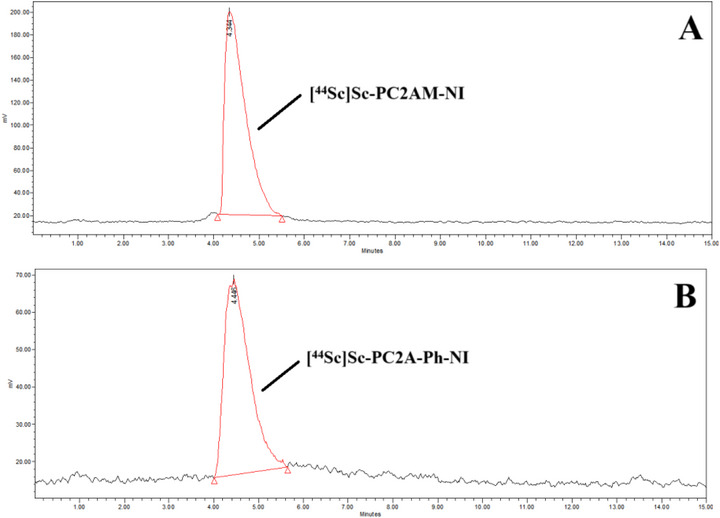
Radio‐HPLC chromatograms of [^44^Sc]]Sc(PC2AM‐NI)]^+^ A) and [^44^Sc][Sc(PC2A‐Ph‐NI)]^+^ radio‐HPLC B).

Following promising physico‐chemical studies, we have carried out ^44^Sc labelling of NI‐containing PC2AM‐NI and PC2A‐Ph‐NI ligands to explore their possible application for tumor hypoxia detection by PET imaging. The ligands were radiolabeled with cyclotron produced ^44^Sc from natural calcium targets with proton irradiation via the ^44^Ca(p, n)^44^Sc reaction.^[^ ^59^ ^]^ The separation and purification of ^44^Sc nuclide, as well as the radiolabeling of the PC2AM‐NI and PC2A‐Ph‐NI ligands, were carried out using our previously reported method.^[^ ^33^ ^]^ Briefly, the irradiated Ca target was dissolved in HCl (3 M, 4 mL), and the ^44^Sc(III) was separated from the solution with an extraction chromatographic resin based on *N,N,N“,N”*‐tetra‐n‐octyldiglycolamide (DGA resin, Triskem). Radiolabeling of ligands with [^44^Sc]ScCl_3_ solution was performed in NH_4_OAc/HOAc puffer (3 M, pH 4) at 95 °C for 15 minutes. The ^44^Sc(III)‐labeled complexes were purified by solid‐phase extraction (SPE) using a reverse‐phase cartridge (Sep‐Pak C18 Plus Light, Waters) to remove unchelated ^44^Sc(III) and the buffer. The radiochemical purity of the labeled complexes was analyzed with radio‐HPLC. The radiolabeling yield was 92% for [^44^Sc][Sc(PC2AM‐NI)]^+^ and >99% for [^44^Sc][Sc(PC2A‐Ph‐NI)]^+^. The radiochemical purity of the labeled complexes was > 99%, which was analyzed by radio‐HPLC (Figure 10). The molar activity for both radiotracers was about 1.1 GBq/µmol, which is similar to the values obtained for ^68^Ga‐labeled complexes [^68^Ga]Ga(DOTA‐NI) ([^68^Ga]4, 4.81 GBq/µmol) and [^68^Ga]Ga(SCN‐DOTA‐NI) ([^68^Ga]5, 7.77 GBq/µmol) by Hoegebazard et al.^[^ ^60^ ^]^

**Figure 11:**
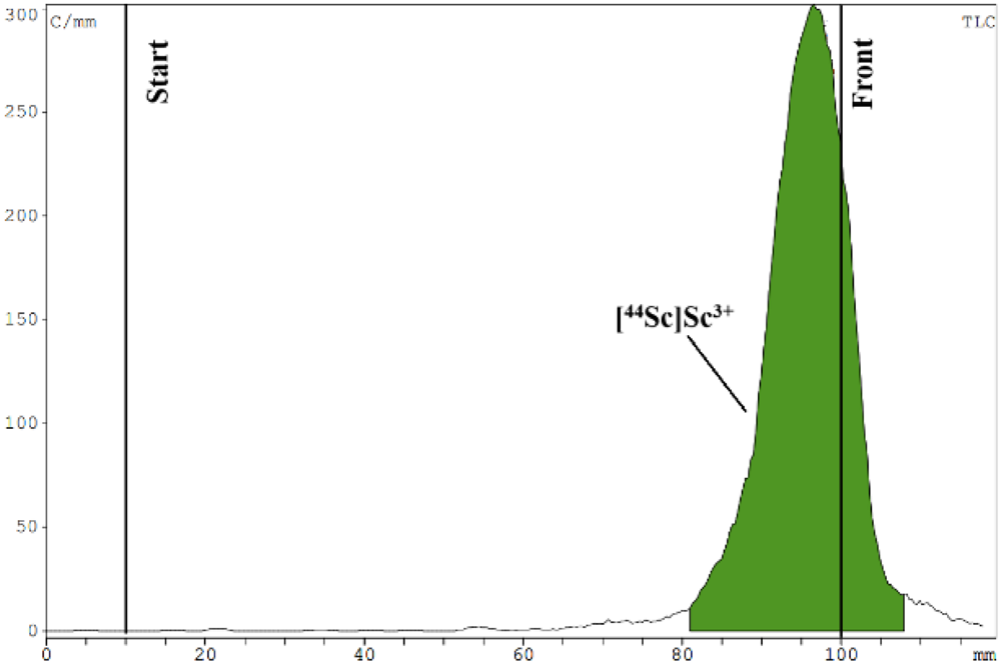
iTLC chromatogram of [^44^Sc]ScCl_3_ solution.

**Figure 12:**
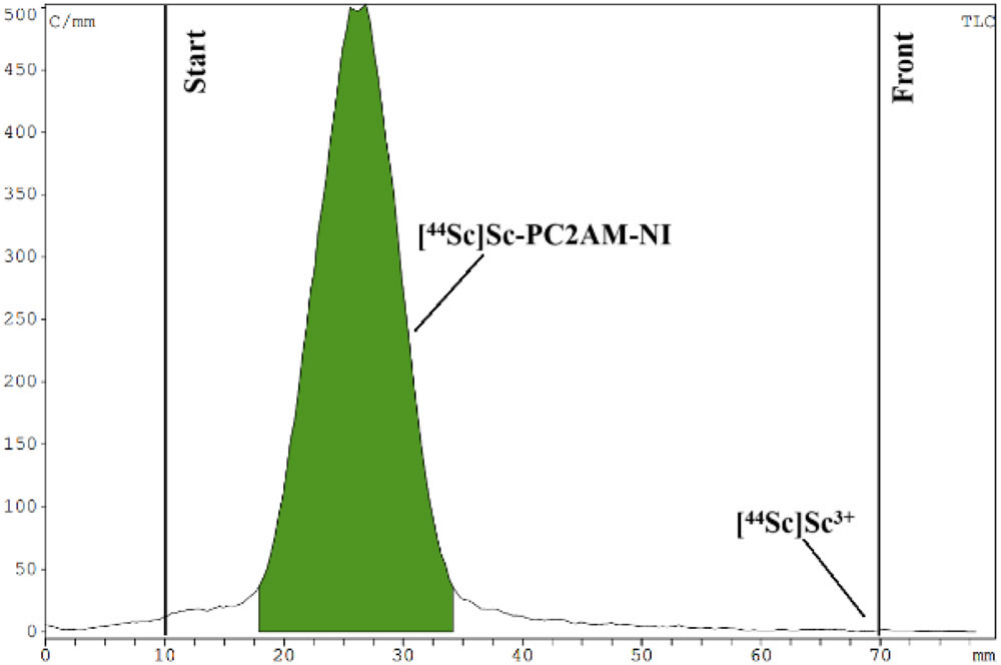
Stability test of [^44^Sc][Sc(‐PC2AM‐NI)]^+^ after 4 hours in rat serum.

**Figure 13:**
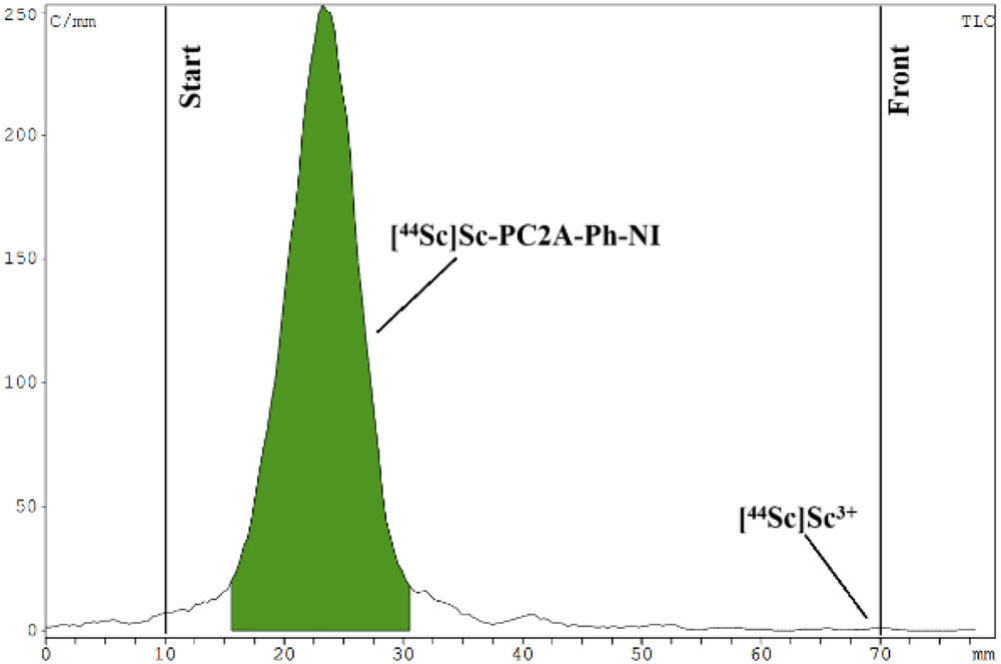
Stability test of [^44^Sc][Sc(PC2A‐Ph‐NI)]^+^ after 4 hours in rat serum.

To evaluate the lipophilicity of labeled compounds, the octanol‐water partition coefficient (log*P*) of the ^44^Sc‐labeled ligands was determined, which was ‐2.44 for [^44^Sc][Sc(PC2AM‐NI)]^+^ and ‐1.25 for [^44^Sc][Sc(PC2A‐Ph‐NI)]^+^ confirming the higher lipophilicity of the latter. Furthermore, the stability and inertness of the purified ^44^Sc‐labeled complexes were investigated in rat serum, the presence of EDTA ligand and endogenous ions. Both radiolabeled complexes were incubated with rat serum, 0.2 M EDTA solution (pH 7.4), and a 1:1 mixture of 0.1 mM ZnCl_2_ and 0.01 mM CuCl_2_ (1 µl) and a 1:1 mixture of 1.02 mM MgCl_2_ and 2.28 mM CaCl_2_ (50 µl) at room temperature for 4 hours, respectively. Samples from the mixtures were analyzed hourly with instant thin‐layer chromatography (iTLC) during the serum stability study, transchelation, and transmetallation tests. The ^44^Sc‐labeled radiopharmaceuticals showed high stability and inertness between the tested conditions for 4 hours. Figures 11, 12, 13 show the iTLC chromatograms of the serum stability test after 4 hours.

These in vitro tests have demonstrated the suitability of PC2AM‐NI and PC2A‐Ph‐NI ligands to stabilize ^44^Sc(III) by complexation for in vivo study of tumor hypoxia by PET imaging as a radiotracer.

### 2.5 In vivo PET and ex vivo animal studies

**Figure 14:**
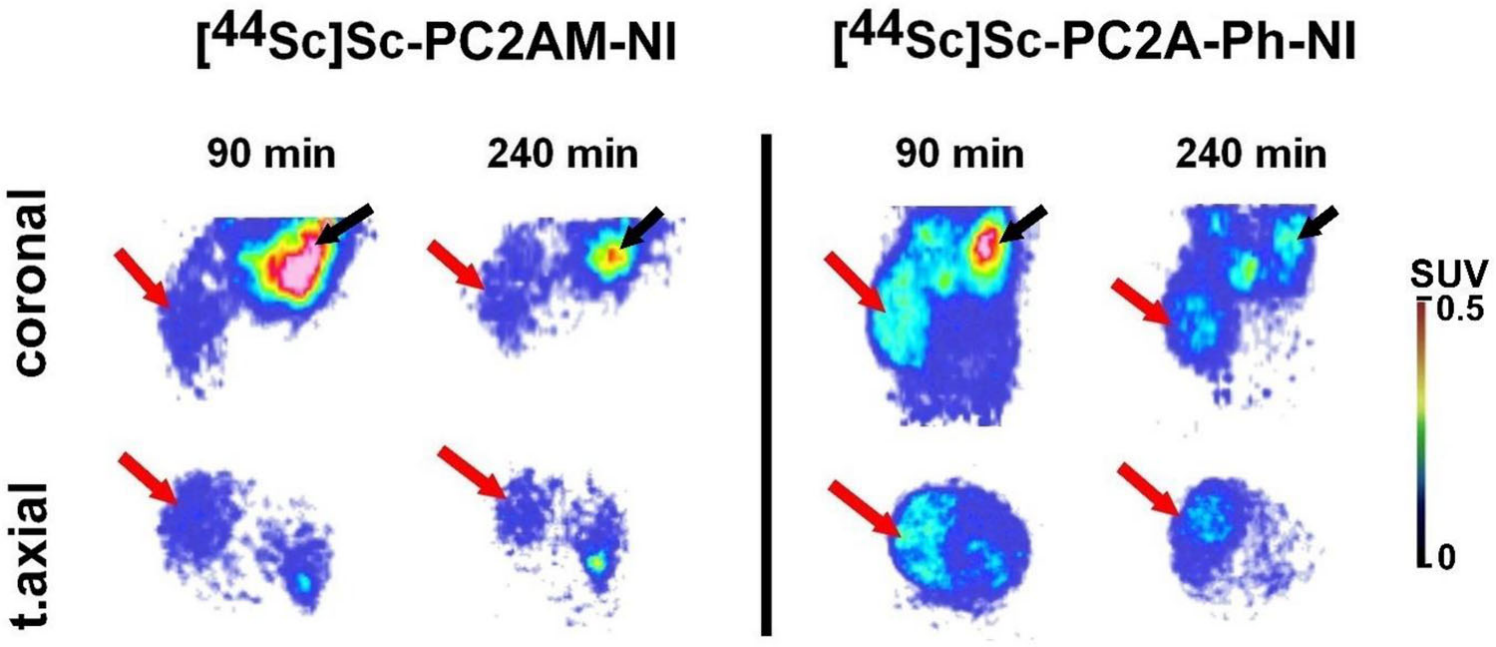
Representative decay‐corrected coronal (upper row) and transaxial (lower row) [^44^Sc][Sc(PC2AM‐NI)]^+^ and [^44^Sc][Sc(PC2A‐Ph‐NI)]^+^ PET images of B16‐F10 tumor‐bearing C57BL/6 mice 90 ‐ and 240‐minutes post tracer injection and 14 days after tumor cell inoculation. Red arrows: B16‐F10 tumor. Black arrows: kidney.

The in vivo tumor‐homing capability and distribution characteristics of [^44^Sc][Sc(PC2AM‐NI)] and [^44^Sc][Sc(PC2A‐Ph‐NI)]^+^ radiopharmaceuticals were described by the PET images of the B16‐F10 tumor models (Figure 14). The qualitative analysis of the decay‐corrected PET images (Figure 14) revealed that the subcutaneously growing B16‐F10 tumors were clearly detectable with both pharmacons (Figure 14, red arrows). Although the background activities were lower for [^44^Sc][Sc(PC2AM‐NI)]^+^ than for the PC2A‐Ph‐NI counterpart, as for the tumors, higher uptakes were observed by using [^44^Sc][Sc(PC2A‐Ph‐NI)]^+^ compared to [^44^Sc][Sc(PC2AM‐NI)]^+^ at each investigated time point. In addition, the tumor uptake showed a gradual decrease over time in the case of both labeled compounds. All these visual observations were confirmed by quantitative PET data, presented in Table 4.

**Table 4:** Quantitative SUV data of [^44^Sc][Sc(PC2AM‐NI)]^+^ and [^44^Sc][Sc(PC2A‐Ph‐NI)]^+^ radiopharmaceuticals in B16‐F10 tumor‐bearing mice. n  =  5 mice/radiopharmacon/time point. *SUV: standardized uptake value; T/M: tumor‐to‐muscle ratio*.

	[^44^Sc][Sc(PC2AM‐NI)]^+^		[^44^Sc][Sc(PC2A‐Ph‐NI)]^+^	
	**90 minutes**	**240 minutes**	**90 minutes**	**240 minutes**
**B16‐F10 SUVmean**	0.08 ± 0.02	0.04 ± 0.02	0.14 ± 0.03	0.10 ± 0.03
**muscle SUVmean**	0.02 ± 0.01	0.01 ± 0.01	0.05 ± 0.01	0.01 ± 0.01
**T/M ratio**	4.04 ± 0.02	4.02 ± 0.02	2.81 ± 0.04	10.12 ± 0.24

**Figure 15:**
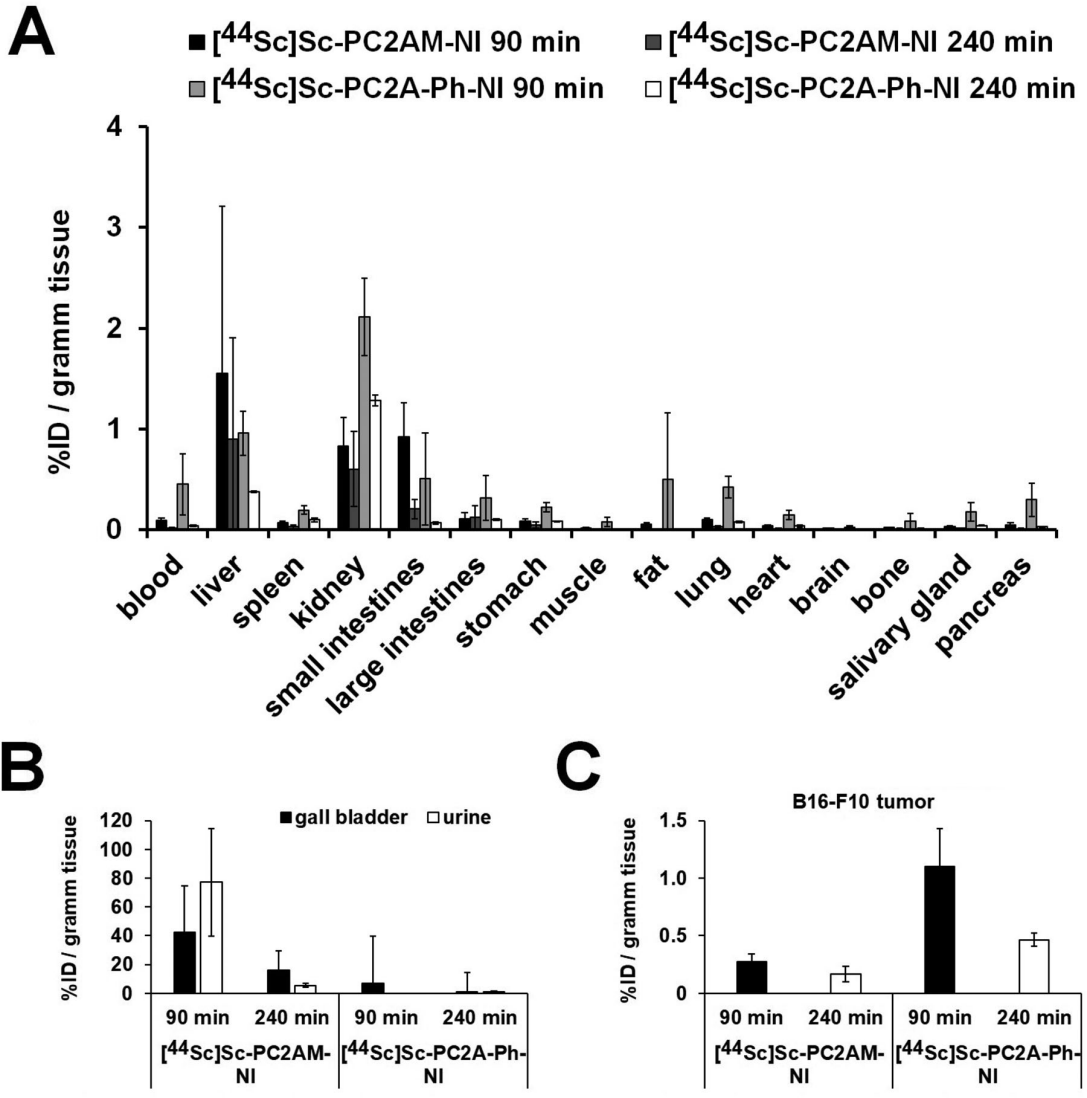
Biodistribution data of [^44^Sc][Sc(PC2AM‐NI)]^+^ and [^44^Sc][Sc(PC2A‐Ph‐NI)]^+^ in healthy control (**Panel A** and **Panel B**) and B16‐F10 melanoma cell‐bearing (**Panel C**) mice 90 ‐ and 240 minutes post injection and 14 days after cancer cell inoculation. n  =  5 tumor‐bearing mice/radiopharmacon/time point and n  =  3 healthy control mice/radiopharmacon/time point.

The biodistribution of [^44^Sc][Sc(PC2AM‐NI)]^+^ and [^44^Sc][Sc(PC2A‐Ph‐NI)]^+^ was investigated by ex vivo studies (Figure 15; Table 5). Indicating hepatobiliary and renal elimination, high %ID/g data were observed for the liver and the kidneys (Table 5). In contrast, the other abdominal and thoracic organs and tissues showed discrete tracer accumulations with both molecules (Figure 15, panel A; Table 5). In accordance with the in vivo SUV data, [^44^Sc][Sc(PC2A‐Ph‐NI)]^+^ showed a higher %ID/g value in the B16‐F10 tumors at both investigated time points than [^44^Sc][Sc(PC2AM‐NI)]^+^ (Figure 15, panel C; Table 5). Biodistribution data of [^44^Sc][Sc(PC2AM‐NI)]^+^ and [^44^Sc][Sc(PC2A‐Ph‐NI)]^+^ in healthy control and B16‐F10 melanoma cell‐bearing mice are summarized in Figure 15 and Table 5.

**Table 5:** *Ex vivo* biodistribution data of [^44^Sc][Sc(PC2AM‐NI)]^+^ and [^44^Sc][Sc(PC2A‐Ph‐NI)]^+^ in healthy control and B16‐F10 melanoma cell‐bearing mice 90, ‐ and 240 minutes post injection and 14 days after cancer cell inoculation. n  =  5 tumor‐bearing mice/radiopharmacon/time point and n  =  3 healthy control mice/radiopharmacon/time point.

	[^44^Sc][Sc(PC2AM‐NI)]^+^		[^44^Sc][Sc(PC2A‐Ph‐NI)]^+^	
	**90 minutes**	**240 minutes**	**90 minutes**	**240 minutes**
**Blood**	0.09 ± 0.02	0.02 ± 0.01	0.45 ± 0.30	0.04 ± 0.01
**Liver**	1.55 ± 1.66	0.90 ± 1.01	0.96 ± 0.22	0.38 ± 0.01
**Spleen**	0.07 ± 0.02	0.03 ± 0.02	0.19 ± 0.04	0.10 ± 0.02
**Kidney**	0.83 ± 0.29	0.60 ± 0.37	2.12 ± 0.38	1.28 ± 0.05
**small intestines**	0.92 ± 0.33	0.20 ± 0.10	0.50 ± 0.46	0.07 ± 0.01
**large intestines**	0.10 ± 0.07	0.12 ± 0.12	0.31 ± 0.22	0.10 ± 0.01
**Stomach**	0.09 ± 0.02	0.04 ± 0.03	0.22 ± 0.05	0.08 ± 0.00
**Muscle**	0.02 ± 0.00	0.00 ± 0.00	0.08 ± 0.05	0.00 ± 0.00
**Fat**	0.05 ± 0.02	0.01 ± 0.00	0.50 ± 0.66	0.00 ± 0.00
**Lung**	0.10 ± 0.02	0.03 ± 0.01	0.42 ± 0.11	0.08 ± 0.01
**Heart**	0.04 ± 0.01	0.01 ± 0.00	0.14 ± 0.05	0.03 ± 0.01
**Brain**	0.01 ± 0.01	0.01 ± 0.00	0.02 ± 0.02	0.00 ± 0.00
**Bone**	0.02 ± 0.00	0.01 ± 0.00	0.08 ± 0.08	0.01 ± 0.00
**salivary gland**	0.03 ± 0.00	0.01 ± 0.01	0.18 ± 0.09	0.04 ± 0.00
**Pancreas**	0.04 ± 0.02	0.01 ± 0.00	0.30 ± 0.17	0.03 ± 0.00
**gall bladder**	42.46 ± 33.31	15.82 ± 13.89	7.17 ± 32.31	0.75 ± 13.89
**Urine**	77.19 ± 37.26	5.44 ± 1.23	106.82 ± 25.31	1.14 ± 0.14
**B16‐F10 tumor**	0.27 ± 0.07	0.17 ± 0.07	1.10 ± 0.33	0.46 ± 0.06

Given the higher tumor uptakes of [^44^Sc][Sc(PC2A‐Ph‐NI)]^+^ with decreasing off‐target activity and related better tumor/noise ratios, we may conclude that the PC2A‐Ph‐NI derivative outperforms the diagnostic capability of [^44^Sc][Sc(PC2AM‐NI)]^+^. The reason for the difference in the behavior of the two ligands is not yet fully understood, as it may arise from differences in ligand structures, the lipophilicity of the complexes, and other factors. Therefore, further studies are required to uncover the exact underlying cause.

## 3 Summary

Rigid pyclen‐based ligands bearing hypoxia‐sensitive NI moieties, PC2A‐Ph‐NI (hexadentate) and PC2AM‐NI (heptadentate), were synthesized and radiolabeled with [^44^Sc]Sc(III) for potential preclinical applications. Their physicochemical properties were investigated using a structurally related model compound, PC2AM^nBu^, obtained in the current study, through pH‐potentiometry, UV‐Vis, and multinuclear NMR spectroscopies. The resulting [Sc(PC2AMnBu)]^+^ complex exhibited high thermodynamic stability (log *K*_ScL_  =  19.53(4); pSc  =  19.32), rapid formation kinetics, and outstanding inertness (*t*_1/2_   =  529 hours in 1 M HClO_4_). Furthermore, the [Sc(PC2AMnBu)]^+^ complex was capable of fluoride binding (log *K*_[Sc(PC2AMnBu)(F)]  _ =  2.5(264, 44, 21834–21848)), with the ternary species showing notable resistance to fluoride ligand exchange (*k*_obs_  =  0.6 s^−1^). These findings underscore the potential of the PC2AM (PC2A‐mono(amide)) ligand platform as a promising ligand scaffold for stable Sc(III) complexation, supporting its suitability for efficient [^44^Sc]Sc(III)‐ and ^18^F‐labeling of NI‐containing ligands. Based on the promising physicochemical properties of the PC2AM^nBu^ ligand, radiolabeling of two NI‐containing ligands with [^44^Sc]Sc(III) was carried out, resulting in a radiolabeling yield of 92% for [^44^Sc][Sc(PC2AM‐NI)]^+^ and > 99% for [^44^Sc][Sc(PC2A‐Ph‐NI)]^+^. The [Sc(PC2AMnBu)]^+^ complex was successfully labeled with ^18^F, yielding 56% radiochemical conversion, thus confirming its suitability as a fluoride carrier for radiochemical applications. The in vitro stability studies of these radiotracers showed that they can be characterized by a high degree of stability and inertness under the tested conditions. In addition, the biodistribution experiments showed exceptional tumor‐targeting ability and favorable T/M ratios for both radiopharmaceuticals, suggesting high diagnostic performance and suitable tracer kinetics for future imaging purposes. Therefore, [^44^Sc][Sc(PC2AM‐NI)]^+^ and [^44^Sc][Sc(PC2A‐Ph‐NI)] have the potential to widen the diagnostic armamentarium of hypoxia‐selective PET probes; moreover, with their [^47^Sc]Sc(III)‐labeled therapeutic counterpart, these theragnostic agents may have future therapeutic implications as well.

## 4 Materials and Methods

### 4.1 Synthesis

Commercial reagents/solvents were purchased from Merck KGaA (Darmstadt, Germany) and Tokyo Chemical Industry (Tokyo, Japan). The purification of 2‐bromo‐*N*‐butylacetamide (3) was performed on a CombiFlash EZ Prep flash chromatograph (#68–5230–026) equipped with a diode array detector. The columns contained silica gel. A Waters Alliance 2690 HPLC system connected with a Waters 996 PDA detector was used to monitor the synthetic reactions and verify the purity of the products. The analytical HPLC is equipped with a Phenomenex C18(2) 150*4.6 mm 5 micron column. The preparative HPLC separations were performed using a YL9100 HPLC system (Korea) equipped with a YL9101S degasser, YL9110S pump, YL9120S UV/VIS detector, and Phenomenex Luna Prep C18(2) 100A 250x21.20 mm 10‐micron column. Sigma‐Aldrich CHROMASOLV Plus acetonitrile and deionized water with 0.005 M trifluoracetic acid were used as solvents for both HPLC techniques. NMR measurements were carried out with Bruker Avance DRX 360 MHz (equipped with a 5 mm QNP probe) and Bruker Avance II 500 MHz (equipped with a 5 mm z‐gradient BBI and TXI probe) spectrometer, the spectra calibrated by the signal of the deuterated solvents. Mass spectrometric characterizations were performed by the Analytical Chemistry Research Group at the Department of Inorganic and Analytical Chemistry, University of Debrecen (Dr. Attila Gáspár, Dávid Ruben Szabó), using a Bruker maXis II UHR ESI‐QTOF MS instrument.

#### 4.1.1 2‐bromo‐*N*‐butylacetamide (3)


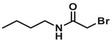


Freshly oven‐dried K_2_CO_3_ (1.0 eq., 13.7 mmol, 1.89 g) was placed in a multi‐neck flask, and 80 mL of dichloromethane was added. Butylamine ([109–73–9], 1.0 eq., 13.7 mmol, 1.00 g) was then added to the mixture. A dropping funnel was attached to the flask, containing a solution of bromoacetyl bromide ([598–21–0], 0.9 eq., 12.3 mmol, 2.48 g) in 30 mL of dichloromethane.

The flask was cooled in an ice–salt bath and flushed with argon, followed by the addition of bromoacetyl bromide solution dropwise to the cooled mixture. The cooling bath was maintained under the flask for one hour while the reaction mixture became opalescent. Upon completion of the reaction, the solid precipitate was filtered through a G3 glass filter and washed with dichloromethane. The combined filtrate was extracted sequentially with 50 mL of water, 50 mL of 5 w/w% citric acid solution, and another 50 mL of water. The organic layer was dried over MgSO_4_, the drying agent was filtered off, and the solvent was removed under reduced pressure.

The crude product was purified by flash chromatography (solvents: hexane, ethyl acetate). The product was obtained as a colorless oil (500 mg, yield: 21%).

^1^H‐NMR (360 MHz, CDCl_3_) δ (ppm): 7.05 (1H, s, ‐N*H*‐), 3.78 (2H, s, ‐C*H_2_*‐), 3.17 (2H, m, ‐C*H_2_*‐), 1.42 (2H, m, ‐C*H_2_*‐), 1.26 (2H, m, ‐C*H_2_*‐), 0.83 (3H, t, ‐C*H_3_*);

^13^C‐NMR (90 MHz, CDCl_3_) δ (ppm): 166.0 (*C * =  O), 39.8, 31.1, 29.1, 19.9 (‐*C*H_2_‐), 13.6 (‐*C*H_3_);

ESI HR‐MS for C_6_H_12_BrNO (m/z, positive mode): [M + H]^+^_calc _ =  194.0175, [M + H]^+^_found _ =  194.0174.

#### 4.1.2 PC2A^Et^AM^nBu^ (5)


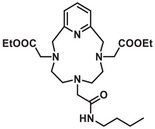


K_2_CO_3_ (3 eq., 3.96 mmol, 329 mg) was added to a three‐neck round‐bottom flask. A solution of 3,9‐PC2AEt (4, 1.0 eq., 0.793 mmol, 300 mg) in 150 mL of anhydrous acetonitrile was added to the flask, and a solution of 2‐bromo‐N‐butylacetamide (3, 1.0 eq., 0.793 mmol, 154 mg) in 50 mL of anhydrous acetonitrile was transferred into an attached dropping funnel. The solution was added dropwise to the stirred reaction mixture at room temperature under an argon atmosphere. Stirring was continued after addition, and the reaction reached completion after 1 hour. The solid precipitate formed during the course of the reaction was filtered off using a G3 glass filter, and the filtrate was evaporated under reduced pressure using a rotary evaporator. The crude product was purified by preparative HPLC (solvent A: 5 mM TFA in water; solvent B: acetonitrile; gradient: 65% A to 40% A over 6 minutes). The product was obtained as a pale yellow oil (280 mg, yield: 72%).

^1^H‐NMR (500 MHz, CD_3_CN) δ (ppm): 7.87 (1H, s, ‐N*H*‐), 7.77 (1H, t, arom.), 7.20 (2H, d, arom.), 4.19 (4H, q, ‐C*H_2_*‐), 4.06 (2H, m, ‐C*H_2_*‐), 3.99 (2H, m, ‐C*H_2_*‐), 3.96 (2H, m, ‐C*H_2_*‐), 3.79 (2H, s, ‐C*H_2_*‐), 3.66 (4H, m, ‐C*H_2_*‐), 3.39 (2H, m, ‐C*H_2_*‐), 3.25 (4H, q, ‐C*H_2_*‐), 3.16 (2H, m, ‐C*H_2_*‐), 1.54 (2H, m, ‐C*H_2_*‐), 1.39 (2H, m, ‐C*H_2_*‐), 1.27 (6H, t, ‐CH_3_), 0.97 (3H, t, ‐CH_3_);

^13^C‐NMR (500 MHz, CD_3_CN) δ (ppm): 172.2 (2C, *C * =  O), 165.3 (*C * =  O), 160.8 (2C, *C_q_* arom.), 139.2 (*C* arom.), 121.7 (2C, *C* arom.), 61.4, 58.2, 58.1, 53.6, 51.3 (2C, ‐*C*H_2_‐), 47.3, 39.7, 32.0, 20.7 (1C, ‐*C*H_2_‐), 14.5 (2C, ‐*C*H_3_), 14.0 (1C, ‐*C*H_3_);

ESI HR‐MS for C_25_H_41_N_5_O_5_ (m/z, positive mode): [M + H]^+^_calc _ =  492.3180, [M + H]^+^_found _ =  492.3180.

#### 4.1.3 PC2AM^nBu^ (6)


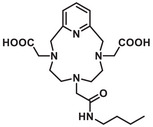


The protected macrocycle (5, 1.0 eq., 0.793 mmol, 390 mg) was dissolved in 40 mL of anhydrous ethanol in a flask. Solid NaOH (4.0 eq., 3.17 mmol, 127 mg) was added to the reaction mixture, and the solution was stirred at room temperature for 2 hours. The solvent was removed under reduced pressure using a rotary evaporator, and the crude product was purified by preparative HPLC (solvent A: 5 mM TFA in water; solvent B: MeCN; gradient: 95% A to 75% A over 8 minutes). The product was obtained as a pale yellow oil (159 mg, yield: 46%).

^1^H‐NMR (360 MHz, CD_3_OD) δ (ppm): 7.85 (1H, t, arom.), 7.32 (2H, d, arom.), 4.95 (4H, s, ‐C*H_2_*‐), 4.36 (4H, s, ‐C*H_2_*‐), 3.88 (2H, s, ‐C*H_2_*‐), 3.86 (4H, s, ‐C*H_2_*‐), 3.40 (4H, s, ‐C*H_2_*‐), 3.26 (2H, t, ‐C*H_2_*‐), 1.52 (2H, m, ‐C*H_2_*‐), 1.38 (2H, m, ‐C*H_2_*‐), 0.96 (3H, t, ‐C*H_3_*); ^13^C‐NMR (360 MHz, CD_3_OD) δ (ppm): 172.7 (2C, *C * =  O), 168.5 (*C * =  O), 158.2 (2C, *C*_q_ arom.), 140.2 (*C* arom.), 122.4 (2C, *C* arom.), 59.2, 57.8, 53.7, 53.0, 40.31, 32.3, 21.1 (2C, ‐*C*H_2_‐), 50.8 (1C, ‐*C*H_2_‐), 14.0 (1C, ‐*C*H_3_); ESI HR‐MS for C_21_H_33_N_5_O_5_ (m/z, positive mode): [M + H]^+^_calc _ =  436.2554, [M + H]^+^_found _ =  436.2554; Analytical HPLC: *t*_R_  =  4.540 minutes (Eluent A: 5 mM TFA in water, Eluent B: MeCN; gradient: 95% A to 10% A over 12 minutes). The NMR and MS spectra, as well as the analytical chromatograms of the ligands studied, are provided in the Supporting Information (Figures S1‐S12).

### 4.2 Equilibrium studies

The protonation constants of the ligands, as well as the concentrations of the ligand stock solutions, were determined by pH‐potentiometric method using a Metrohm 785 DMP Titrino automatic titrator using a Metrohm 6.00234.100 combined glass electrode. The titrations were carried out in the pH range 1.75–11.80 and the pH electrode was calibrated by a two‐point calibration routine with the use of 0.05 M KH‐phthalate (pH  =  4.005) and 0.01 M borax (pH  =  9.177 buffers. The samples were stirred at 25 °C (±0.1 °C) and N_2_ gas bubbled into the system to ensure inert conditions and to eliminate the effect of the presence of CO_2_. For the calculation of [H^+^] from the measured pH values, the method proposed by Irving et al. was applied.^[^ ^61^ ^]^ For this, a 0.01 M HCl solution was titrated with the standardized NaOH solution in the presence of 0.15 M NaCl ionic strength at 25 °C. The differences between the measured (pH_read_) and calculated pH (‐log[H^+^]) values were used to obtain the equilibrium H^+^ concentration from the pH_meas_ values during the titrations. By fitting the resulting titration curves (pH – V (NaOH) data pairs) with the PSEQUAD program, the protonation constants of the ligands were determined.^[^ ^47^ ^]^ The titration of the [Sc(PC2AM^nBu^)]^+^ with NaOH was performed with the same method by applying very similar conditions.

The stability constant of [Sc(PC2AM^nBu^)]^+^ and [Sc(PCTA)] complexes was studied using “out‐of‐cell” (batch) samples using ^45^Sc and ^1^H NMR spectroscopy. The NMR spectra were obtained using a Bruker Avance II DRX spectrometer (9.4 T), equipped with Bruker Variable Temperature Unit, Bruker Cooling Unit, and a 5 mm BB inverse *z* gradient probe. For the investigation of [Sc(PC2AMnBu)]⁺, twelve samples (c_Sc(III)_  =  3.00 mM, c_ligand_  =  3.06 mM, in the pH range of –log[H^+^] = 0.1–1.3; I  =  1.0 M NaCl/HCl) were prepared in NMR tubes and kept at 25 °C for one week to reach equilibrium. During this period, ^1^H and ^45^Sc NMR measurements were performed daily to monitor changes in signal intensities. No significant changes were detected after one week, even in the most acidic sample. The intensities of the free Sc(III) signals were normalized using a standard 50 mM ScCl_3_ solution containing 1 M HCl (20% D_2_O). For the determination of the stability of the [Sc(PCTA)] complex, eleven “batch” samples (c_Sc(III)_  =  4.00 mM, c_ligand_  =  4.08 mM), in the pH range of –log[H^+^] = 0.1–1.25, were prepared. The evaluation of the measured data was performed using the PSEQUAD program.^[^ ^47^ ^]^ Samples were prepared in H_2_O, with 10% D_2_O added for deuterium lock. The actual resonance frequencies for ^1^H, ^13^C, ^19^F, and ^45^Sc are indicated in the text and figure captions. The chemical shifts of ^1^H and ^13^C were calibrated using an external TMS standard, while ^19^F was referenced to CCl_3_F. The ^45^Sc NMR shift was calibrated to 0 ppm using a 5 mM solution of ScCl_3_ in 0.1 M HCl.

### 4.3 Kinetic studies

^45^Sc NMR measurements (97 MHz) were performed on a Bruker DRX 400 (9.4 T) spectrometer equipped with a Bruker VT‐1000 thermocontroller and a BB inverse z‐gradient probe (5 mm). The kinetic experiments were performed at 25 °C in aqueous solution, using D_2_O (10% v/v) as a lock solvent. Typical parameters for 45Sc NMR measurements were used: TD  =  32k, p1  =  15.6 µs (90°), *d*_1_ =  0.1 s. The formation reactions of the [Sc(PC2AM^nBu^)]^+^ and [Sc(PCTA)] complexes were monitored by ^45^Sc NMR spectroscopy in two sample types: one containing 10.0 mM PC2AM^nBu^ and ScCl_3_ / 9.5 mM PC2AM^nBu^ and ScCl_3_. In both cases, the total acid concentration corresponded to a pH of 1.28. Obtained monoexponential curves (signal area of free Sc(III) vs. time) were fitted using Scientist software ^[^ ^62^ ^]^ to determine the formation rate.

Kinetic studies of the dissociation of the [Sc(PC2AMnBu)]^+^ and [Sc(PCTA)] complexes were carried out by examining the dependence of the dissociation rates on acid concentration. Spectrophotometric measurements were conducted on a JASCO V‐760 spectrophotometer at λ = 280 nm. The concentration of the complex in the sample was 0.30 mM, and HClO_4_ was applied in concentrations ranging from 1.6 to 2.6 M. The evaluation of the measured data was performed with Scientist software ^[^ ^62^ ^]^ to determine the *k*_0_ (spontaneous) and *k*_1_ (acid‐assisted) reaction rates.

### 4.4 Radiochemistry

#### 4.4.1 General

All solvents and reagents that were used for the radiochemical experiments possessed high purity to avoid metallic contamination. Ultrapure (u.p.) water and u.p. HCl were purchased from Carl Roth, and absolute ethanol was purchased from Merck. The ^44^Sc radioisotope was prepared in GE PETtrace cyclotron at the Department of Nuclear Medicine and Translational Imaging, Institute of Medical Imaging, University of Debrecen, Hungary. Activity measurements were carried out with a CAPINTEC CRC‐15PET dose calibrator and a Perkin Elmer Packard Cobra gamma counter. Radio‐HPLC was performed on Waters 2695 Alliance HPLC using Kinetex XB‐C18 2.6 µm (50 × 4.60 mm) column. Radio‐TLC was performed on iTLC paper (Waters) and analyzed using MiniGita TLC‐Scanner with GINA‐Star TLC software. DGA resin was purchased from Triskem International. The purification of the labeled compounds was carried out with a Sep‐Pak C18 Light Plus cartridge (Waters).

#### 4.4.2 Production of [^44^Sc]Sc isotope for radiolabeling

[^44^Sc]Sc nuclide was produced by cyclotron and purified according to the previously published procedure.^[^ ^33^ ^]^ It was performed according to the following protocol, 120 mg of natural calcium (99.99%) was pressed into a pellet and pushed into the cavity of an aluminum target holder. After 60 minutes of irradiation with 30 µA beam current, the irradiated Ca disc was dissolved in 3 M u.p. HCl (4 mL), and the solution was transferred onto a DGA column containing 70 mg resin preconditioned with 3 M u.p (3 mL) HCl. The DGA column was washed with 3 mL 3 M u.p. HCl and 3 mL 1 M HNO_3_ and eluted with 2 mL 0.1 M u.p. HCl in 200 µL fractions. Based on radioactivity measurements, the fractions with the highest ^44^Sc isotope content were collected.

#### 4.4.3 Radiolabeling of PC2AM‐NI and PC2A‐Ph‐NI ligands using [^44^Sc]Sc isotope

100 µL of [^44^Sc]ScCl_3_ solution (∼150 MBq) was transferred into an Eppendorf tube, then 250 µL of NH_4_OAc buffer (3 M, pH 4) and 60 µL aq. stock solution of PC2AM‐NI and PC2A‐Ph‐NI (1 mg/mL) ligands were added, respectively. The mixture was heated at 95 °C for 15 minutes and then passed through a preconditioned SPE cartridge (Sep‐Pak C18 Plus Light). After purging the cartridge with 1 mL of water, the labeled compound was eluted with 250 µL of ethanol and concentrated, and then the labeled product was dissolved in 100 µL of saline. The radiochemical purity of the ^44^Sc‐labeled complexes was determined with radio‐HPLC using Kinetex XB‐C18 2.6 µm (50 × 4.60 mm) column and eluent A: oxalic acid (0.01 M, pH 3), eluent B: acetonitrile. The flow rate was 1 mL/min, and gradient was the following: 0 minutes: 100% A, 1 minutes: 100% A, 10 minutes: 100% B, 10.1 minute: 100% A.

#### 4.4.4 Serum stability study of [^44^Sc][Sc(PC2AM‐NI)]^+^ and [^44^Sc][Sc(PC2A‐Ph‐NI)]^+^ in rat serum

50 µl aqueous solution of both radiolabeled complex (∼3 MBq) was added to 450 µl of rat serum. The mixtures were incubated at room temperature and analyzed at 1, 2, 3, and 4 hours by radioTLC using iTLC paper developed with a 0.5 M citrate solution (pH 5.5).

#### 4.4.5 EDTA challenge test of [^44^Sc][Sc(PC2AM‐NI)]^+^ and [^44^Sc][Sc(PC2A‐Ph‐NI)]^+^

50 µl aqueous solution of both radiolabeled complexes (∼3 MBq) was incubated with 50 µl of 0.2 M EDTA (pH 7.4) solution at room temperature, and samples were taken at 1, 2, 3, and 4 hours. The radiochemical purity of the samples from mixtures was determined with the radio‐iTLC method mentioned above.

#### 4.4.6 Metal challenge test of [^44^Sc][Sc(PC2AM‐NI)]^+^ and [^44^Sc][Sc(PC2A‐Ph‐NI)]^+^

49 µL aqueous solution of both radiolabeled complexes (∼3 MBq) was incubated with a 1:1 mixture of 0.1 mM ZnCl_2_ and 0.01 mM CuCl_2_ (1 µL), and a 1:1 mixture of 1.02 mM MgCl_2_ and 2.28 mM CaCl_2_ (50 µL) at room temperature, respectively. The radiochemical purity of the samples from mixtures at 1, 2, 3, and 4 hours was determined by the radio‐iTLC method mentioned above.

#### 4.4.7 Determination of the octanol‐water partition coefficient (logP) of [^44^Sc][Sc(PC2AM‐NI)]^+^ and [^44^Sc][Sc(PC2A‐Ph‐NI)]^+^

50 µL aqueous solution of both radiolabeled complexes (∼3 MBq) was added to 450 µl water and 500 µl of octanol. The mixture was shaken with a vortex shaker (5 minutes, 1000 rpm) and centrifuged (5 minutes, 10 000 rpm). 3x20 µl from the octanol and 3x20 µl from the water were pipetted into test tube, respectively. The radioactivity of the samples was measured with a gamma detector.

### 4.5 Biology

#### 4.5.1 Cancer cell lines and mouse models

The B16‐F10 mouse melanoma cell line (MC1‐R positive) was the kind gift of the Department of Immunology, Faculty of Medicine, University of Debrecen (Debrecen, Hungary). The cells were routinely cultivated according to the manufacturer's protocols using Dulbecco's Modified Eagle's medium (DMEM, Merck Life Science Ltd., Budapest, Hungary) supplemented with 1% (v/v) MEM Non‐Essential Amino Acid solution (Merck Life Science Ltd.), 1% MEM Vitamins solution (Merck Life Science Ltd.), 10% Fetal Bovine Serum (FBS, GIBCO Life Technologies, Billings, MT, USA), and 1% Antibiotic and Antimicotic solution (Merck Life Science Ltd.) at 37 °C in a humidified atmosphere containing 5% CO_2_. For tumor induction the cells were used at 85% confluence. Cell viability was determined by applying trypan blue exclusion assay. Those cells were used for subsequent experiments where the staining confirmed that more than 90% of them were viable.

All animal experiments confirmed to all applicable sections of the Hungarian Laws and the directions and regulations of the European Union, and the in vivo studies have been ethically approved by the Ethics Committee for Animal Experimentation of the University of Debrecen (license number: 16/2020/DEMÁB). C57BL/6 male mice (n  =  32, older than 12 weeks, approx. 20 grams) obtained from Charles River Animalab Ltd. (Budapest, Hungary) were used for in vivo PET studies and biodistribution assays. To induce tumor models, 20 mice were subcutaneously transplanted with approximately 3x10^6^ tumor cells (in 100 µL physiological saline) at the left shoulder area. Tumor growth was regularly monitored by using caliper measurements. During tumor generation inhalation anesthesia was induced using 3% isoflurane (Forane, AbbVie, Chicago, IL, USA), 0.4 L/min O_2_, and 0.8 L/min N_2_O.

#### 4.5.2 In vivo PET studies

Preclinical PET studies were performed on the MiniPET‐II scanner of the Department of Nuclear Medicine and Translational Imaging, Institute of Medical Imaging, Faculty of Medicine, University of Debrecen (Debrecen, Hungary). Fourteen days post tumor induction, tumor‐bearing mice (n  =  20) were anesthetized (see above) and tail vein injected with approximately 5–7 MBq of [^44^Sc][Sc(PC2AM‐NI)]^+^ or [^44^Sc][Sc(PC2A‐Ph‐NI)]^+^. After a 90 ‐ and a 240‐minutes awake incubation period, the mice were re‐anesthetized and 20‐minutes static PET scans were taken. To quantify radiopharmaceutical uptake, followed by image reconstruction (3D ordered‐subsets expectation‐maximization algorithm (3D‐OSEM)), volumes of interest (VOIs) were manually outlined on the tumors and on the hindlimb muscle tissue as a reference region. Using a dedicated imaging analysis software: BrainCAD image analysis software (version: 1.12‐bulid3), mean standardized uptake values (SUVmean) were determined with the following equation: SUV = [VOI activity (Bq/mL)]/[injected activity (Bq)/animal weight (g)]. To assess image contrast, tumor‐to‐off‐target ratios (tumor‐to‐muscle ratio/T/M) were also calculated.

#### 4.5.3 *Ex vivo* biodistribution studies

To evaluate the organ distribution of the novel ^44^Sc‐labeled radiotracers, ex vivo biodistribution experiments were additionally performed. Healthy control and tumor‐bearing mice were administered with approximately 5–7 MBq of [^44^Sc][Sc(PC2AM‐NI)]^+^ or [^44^Sc][Sc(PC2A‐Ph‐NI)]^+^ and at 90 ‐ and 240‐minutes post injection, all of them were put to death. Organs and tissues of interest were collected, weighed wet, and γ‐counted (Perkin‐Elmer Packard 406 Cobra, Waltham, MA, USA). After decay correction, the radioactivity of each tissue and organ was calculated as %ID/g.

#### 4.5.4 Statistical Analyses

Data were analyzed with the MedCalc 18.5 commercial software package (MedCalc 18.5, MedCalc Software, Mariakerke, Belgium). Two‐tailed t‐tests, two‐way ANOVA, or the Mann‐Whitney U‐test were used for statistical analysis. Statistics for in vivo and ex vivo data are expressed as mean ± SD. *P* values of less than 0.05 were considered statistically significant.

## Conflict of Interest

The authors declare no conflict of interest.

## Supporting information



Supporting Information

## Data Availability

The data that support the findings of this study are available from the corresponding author upon reasonable request.
