# Supplementary material for: Sc(III) Complexes of Pyclen Derivative Ligands as Probes for Hypoxia: Synthesis, Chemical Characterization, 44Sc‐Radiolabeling, and Preclinical Assessment
Source: Chemistry. 2025 Nov 18;31(72):e02763. doi: 10.1002/chem.202502763 (PMC12731536; doi:10.1002/chem.202502763)
Supplement: Supplementary file 1 — Supporting Information [file CHEM-31-e02763-s001.docx]

**Supplementary Material**

**Sc(III) complexes formed with pyclen derivative ligands as imaging probes for hypoxia: synthesis, physicochemical characterization and in vivo of assessment**

Tibor Csupász^‡^, Bayar Dahman^‡†%^, Tamás Gyula Gál^‡^, István Kapus^‡†^, Zita Képes^§^, Dániel Szücs^§^, György Trencsényi^§^, Imre Tóth^‡^, Anikó Fekete*^§^ and Gyula Tircsó*^‡^

^‡^ Department of Physical Chemistry, Faculty of Science and Technology, University of Debrecen, Egyetem tér 1, H-4032 Debrecen, Hungary

^†^ Doctoral School of Chemistry, Faculty of Science and Technology, University of Debrecen, Egyetem tér 1, Debrecen H-4032, Hungary

% Department of Chemistry, Faculty of Science, University of Zakho, Zakho 42002, Kurdistan Region, Iraq

^§^ Department of Nuclear Medicine and Translational Imaging, Institute of Medical Imaging, Faculty of Medicine, University of Debrecen, Nagyerdei krt. 98., Debrecen H-4032, Hungary

*To whom correspondence should be addressed

[fekete.aniko@science.unideb.hu](mailto:fekete.aniko@science.unideb.hu) and [gyula.tircso@science.unideb.hu](mailto:gyula.tircso@science.unideb.hu)

**Table of contents**

| **Figure** | **Title** | **Page** |
| --- | --- | --- |
| **Figure S1**. | 360 MHz ^1^H NMR spectrum of the 3,9-PC2AM^nBu^ ligand in CD_3_OD. | 4 |
| **Figure S2**. | 90.55 MHz ^13^C NMR spectrum of the 3,9-PC2AM^nBu^ ligand in CD_3_OD. | 4 |
| **Figure S3**. | ESI HR-MS of the 3,9-PC2AM^nBu^ ligand (in m/z positive mode). | 5 |
| **Figure S4**. | Analytical HPLC of the of the 3,9-PC2AM^nBu^ ligand | 5 |
| **Figure S5**. | 360 MHz ^1^H NMR spectrum of the 3,9-PC2AM-NI ligand in D_2_O. | 6 |
| **Figure S6**. | 90.55 MHz ^13^C NMR spectrum of the 3,9-PC2AM-NI ligand in D_2_O. | 6 |
| **Figure S7**. | ESI HR-MS of the 3,9-PC2AM-NI ligand (in m/z positive mode). | 7 |
| **Figure S8**. | Analytical HPLC of the of the 3,9-PC2AM-NI ligand. | 7 |
| **Figure S9**. | 360 MHz ^1^H NMR spectrum of the 3,9-PC2A-Ph-NI ligand in CD_3_OD. | 8 |
| **Figure S10**. | 90.55 MHz ^13^C NMR spectrum of the 3,9-PC2A-Ph-NI ligand in CD_3_OD. | 8 |
| **Figure S11**. | ESI HR-MS of the 3,9-PC2A-Ph-NI ligand (m/z in positive mode). | 9 |
| **Figure S12**. | Analytical HPLC of the 3,9-PC2A-Ph-NI ligand. | 9 |
| **Figure S13**. | Measured free Sc(III) concentration from ^45^Sc NMR and calculated species distribution curves for the Sc(III) - (PCTA)^3-^ - H^+^ system as a function of-log[H^+^] values. | 10 |
| **Figure S14**. | Relative ligand affinity data (AL/M) calculated for Sc(III) complexes formed with PCTA, DO3AM-NI and PC2AMnBu ligands as a function of pH as proposed by M. Meyer and co-workers. | 10 |
| **Figure S15**. | 400 MHz ^1^H-NMR spectra of [Sc(PC2AAM^nBu^)]^+^ (aromatic region) at different pH | 11 |
| **Figure S16.** | 400 MHz ^1^H-NMR spectra (aromatic region) of [Sc(PC2AAM^nBu^)]^+^ and equilibrium mixture of [Sc(PC2AAM^nBu^)]^+^ and [Sc(PC2AAM^nBu^)(F)]. | 11 |
| **Figure S17.** | 97.20 MHz ^45^Sc-NMR spectrum of the equilibrium mixture of [Sc(PC2AAM^nBu^)]^+^ and [Sc(PC2AAM^nBu^)(F)]. | 12 |
| **Figure S18**. | Kinetic profile of [Sc(PC2A1AM^nBu^)]^+^ formation monitored by ⁴⁵Sc NMR spectroscopy. | 12 |
| **Figure S19**. | Kinetic profile of [Sc(PCTA)] formation monitored by ⁴⁵Sc NMR spectroscopy. | 13 |
| **Figure S20**. | UV spectra of 0.32 mM PC2A1AM^nBu^ ligand and that of 0.27 mM [Sc(PC2A1AM^nBu^)]^+^ complex in 1.0 M HClO_4_. | 13 |
| **Figure S21.** | UV spectra of 0.30 mM PCTA ligand and that of 0.30 mM [Sc(PCTA)] complex in 2.6 M HClO_4_. | 14 |
| **Figure S22.** | Dissociation kinetic profile of [Sc(PC2AAM^nBu^)]^+^ monitored by UV-Vis spectroscopy in acidic medium. | 14 |
| **Figure S23.** | Dissociation kinetic profile of [Sc(PCTA)] monitored by UV-Vis spectroscopy in acidic medium. | 15 |
| **Figure S24.** | 376.4 MHz ^19^F NMR spectrum recorded for the [Sc(PC2AAMnBu)(F)] and [Sc(PC2AAMnBu)(F)] at pH = 5.20. | 15 |
| **Figure S25.** | Selective magnetization transfer (MT) experiment curves for [Sc(PC2AAM^nBu^)(F)]. | 16 |
| **Figure S26.** | HPLC chromatograms of [^44^Sc]]Sc(PC2AM-NI)]^+^with non-radioactive coinjection. | 17 |
| **Figure S27.** | HPLC chromatograms [^44^Sc][Sc(PC2A-Ph-NI)]^+^ with non-radioactive coinjection. | 17 |
| **Figure S28.** | Radio-HPLC chromatogram of crude reaction mixture of [^18^F][Sc(PC2AM^nBu^)(F)] complex. | 19 |
| **Table S1.** | Summary of data used for the calculation of the stability constant, log *K*_[Sc(PC2AM_^nBu^_)(F)]_ by titration of 0.005M [Sc(PC2AM^nBu^)]^+^ parent complex with different amounts of F^-^ (0.005 - 0.01 M concentration range) followed by 376.4 MHz ^19^F NMR. | 19 |


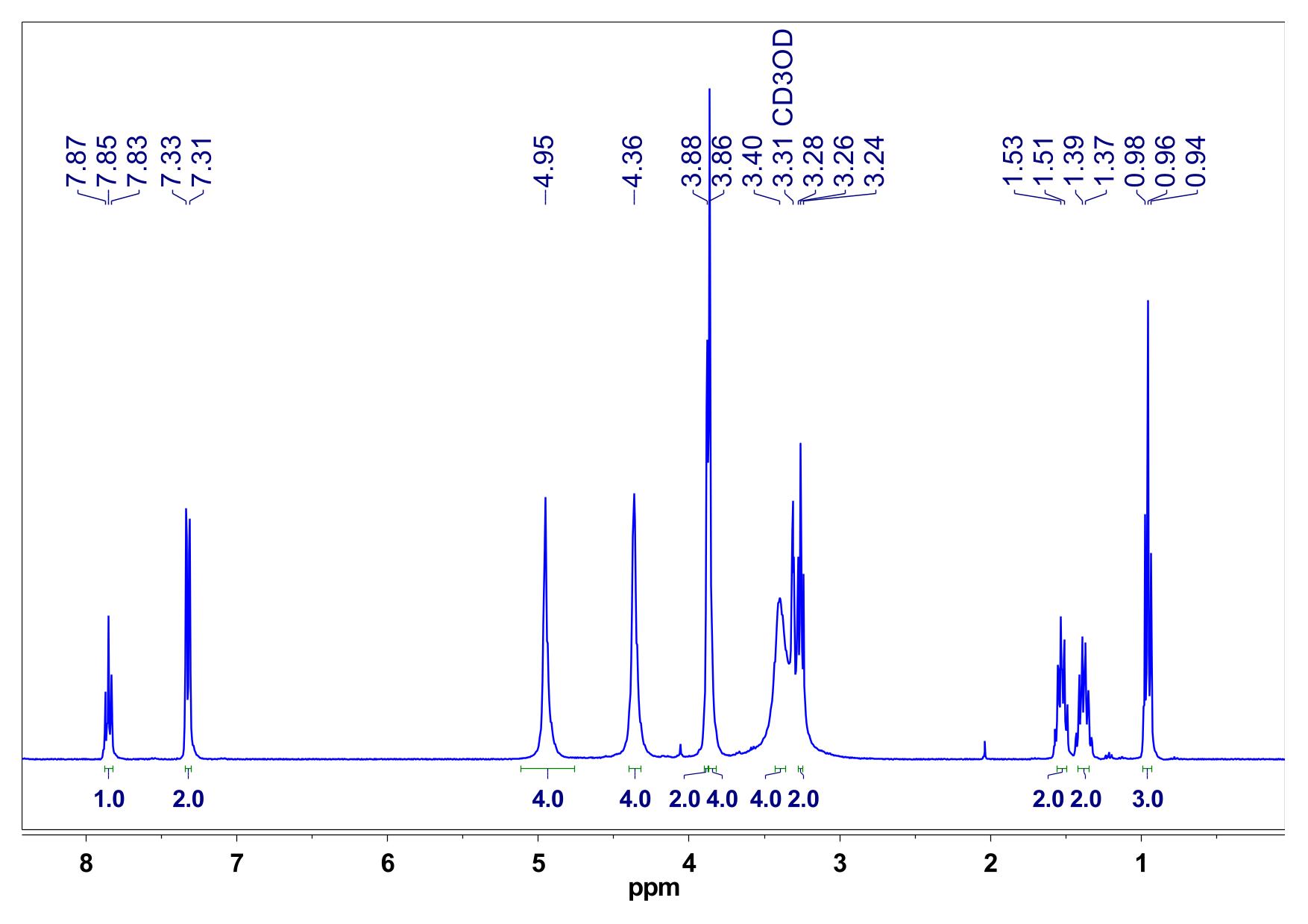


**Figure S1**. 360 MHz ^1^H NMR spectrum of the 3,9-PC2AM^nBu^ ligand in CD_3_OD.


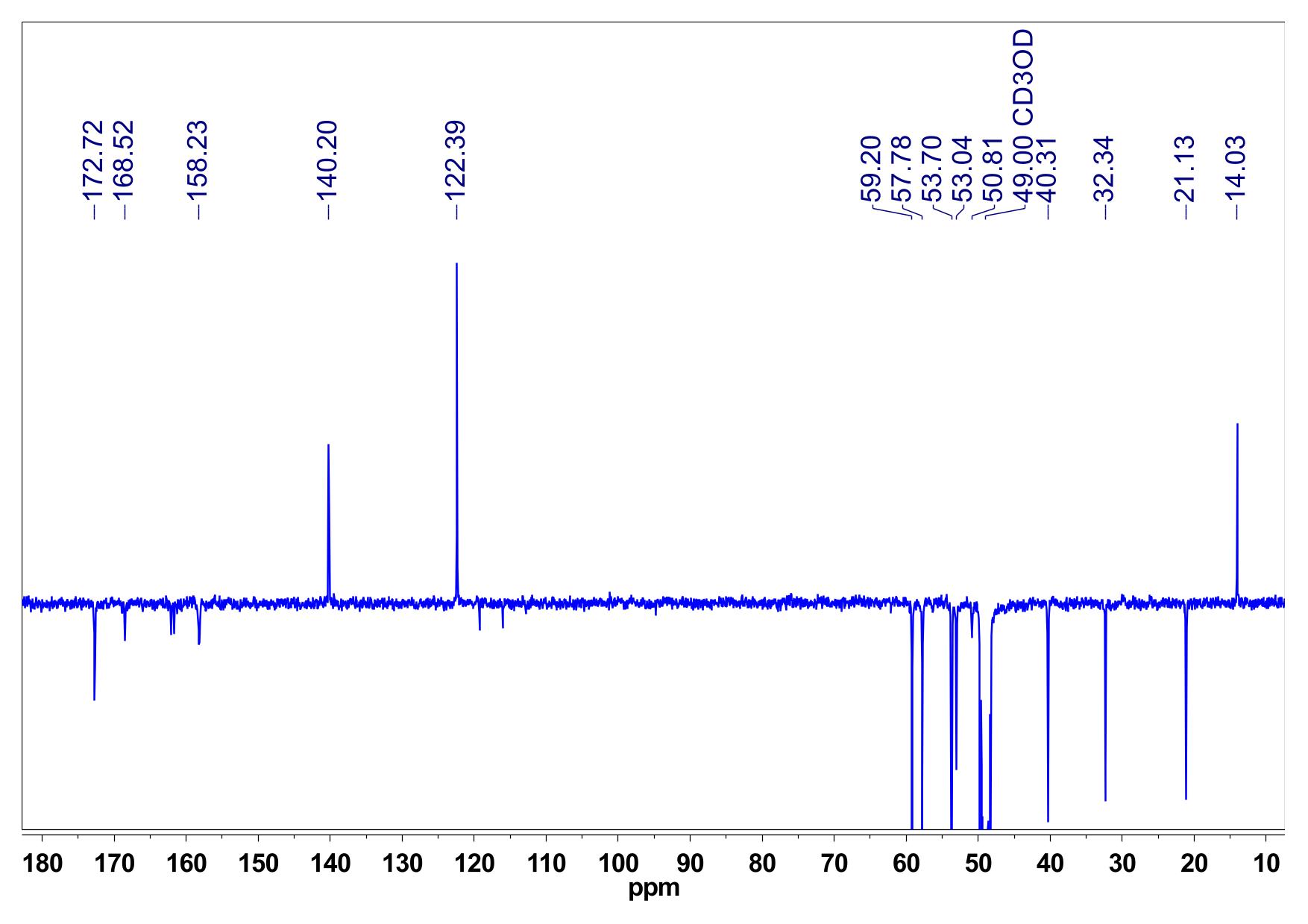


**Figure S2**. 90.55 MHz ^13^C NMR spectrum of the 3,9-PC2AM^nBu^ ligand in CD_3_OD.


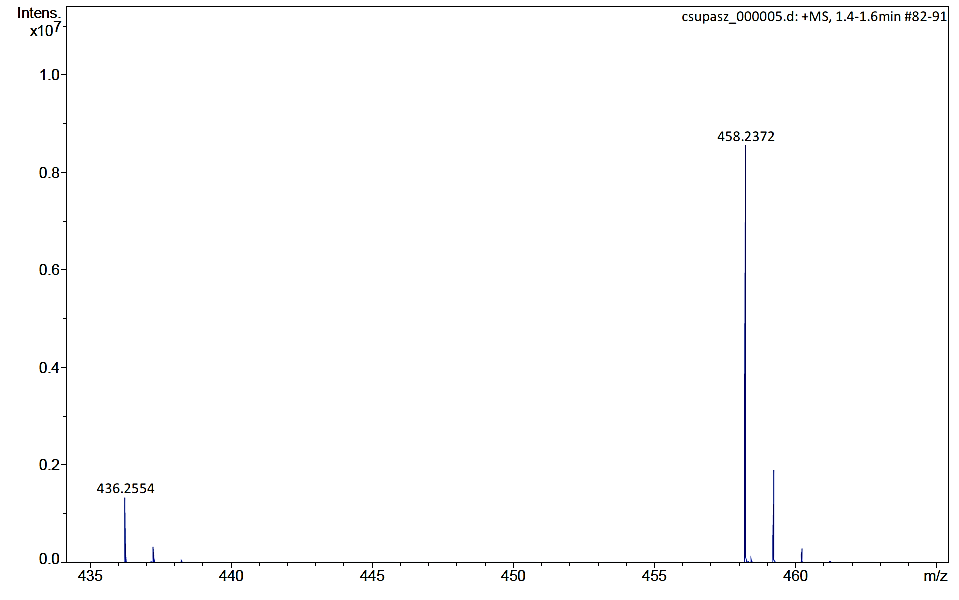


**Figure S3**. ESI HR-MS of the 3,9-PC2AM^nBu^ ligand (for C_21_H_33_N_5_O_5_ in m/z positive mode).


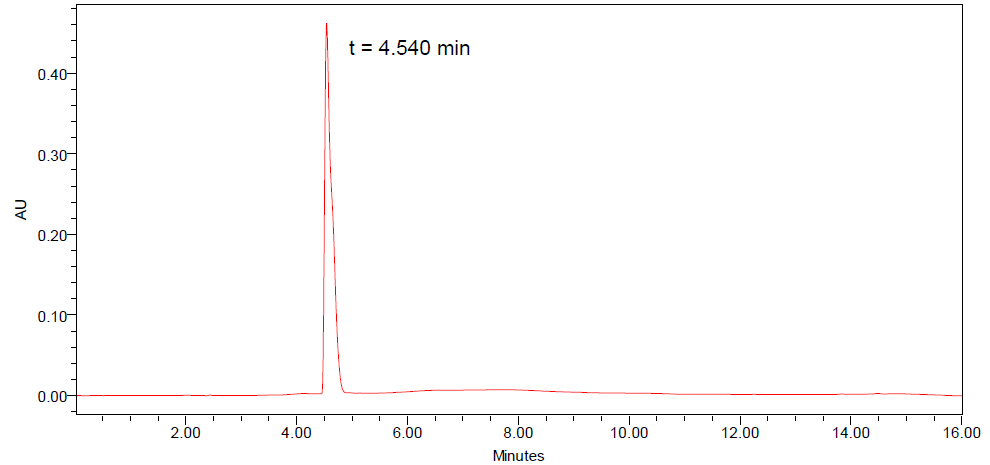


**Figure S4**. Analytical HPLC of the 3,9-PC2AM^nBu^ ligand (*t*_R_ = 4.540 min, Eluent A: 5 mM TFA in water, Eluent B: MeCN; gradient: 95% A to 10% A over 12 minutes).


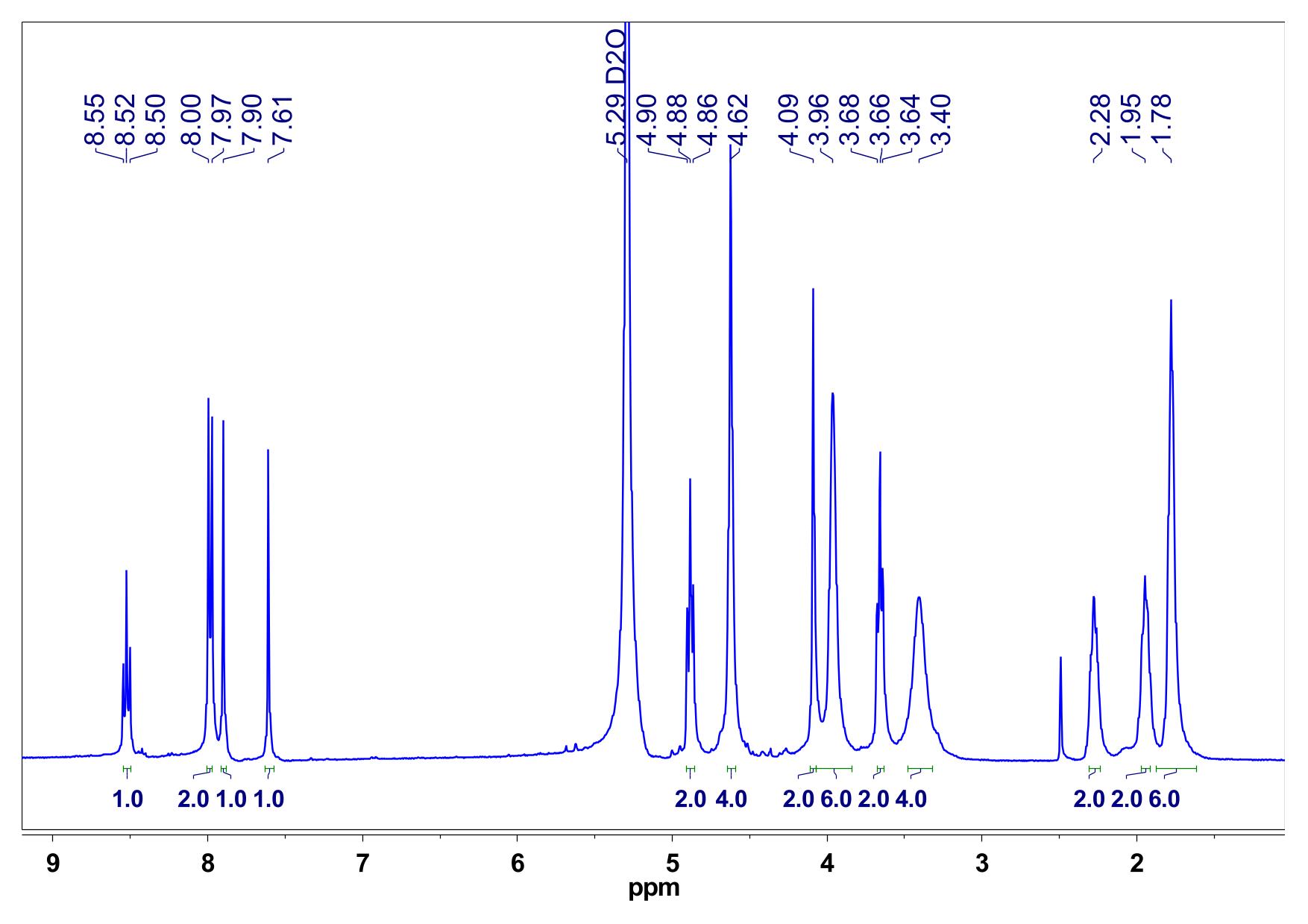


**Figure S5**. 360 MHz ^1^H NMR spectrum of the 3,9-PC2AM-NI ligand in D_2_O.


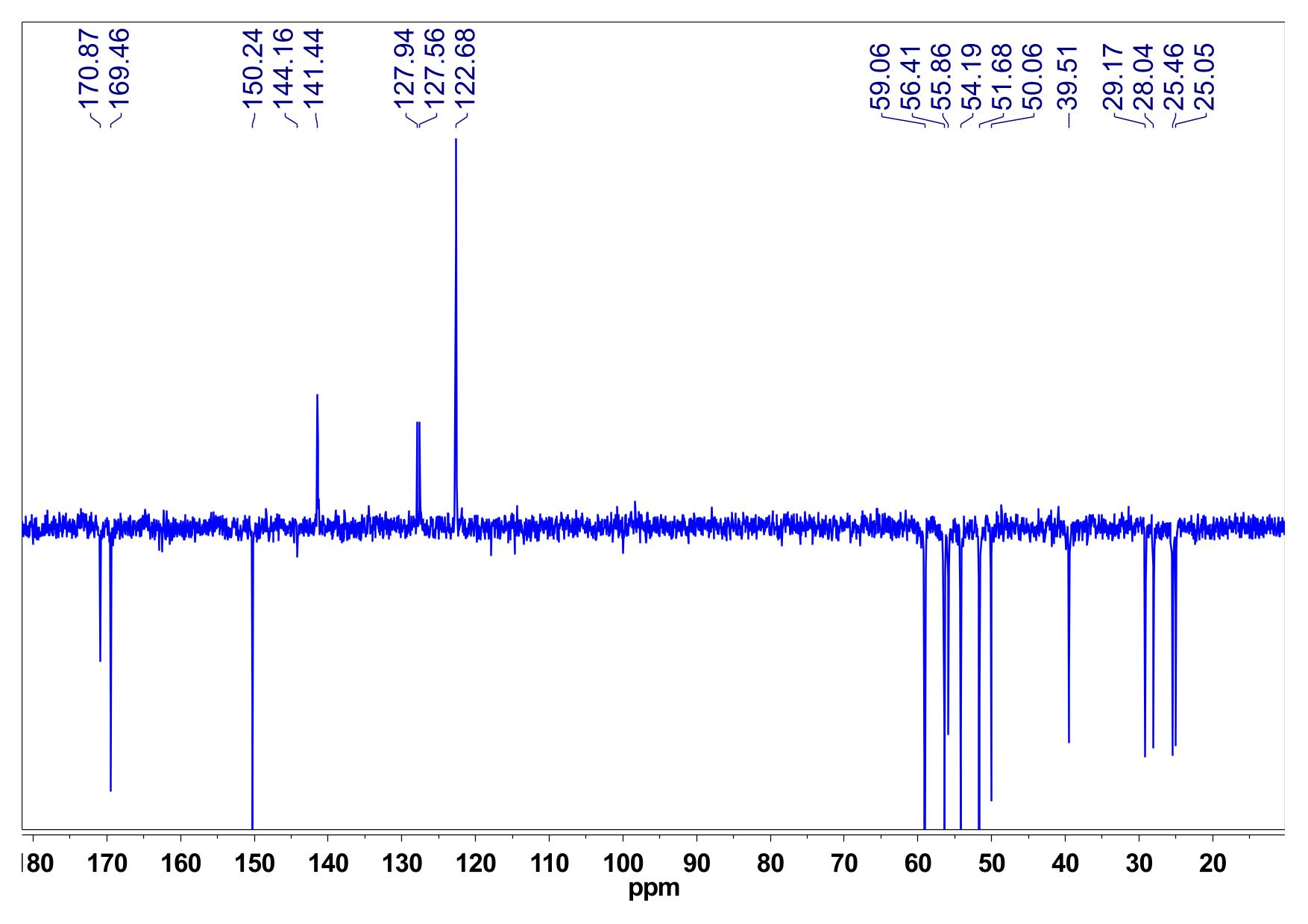


**Figure S6**. 90.55 MHz ^13^C NMR spectrum of the 3,9-PC2AM-NI ligand in D_2_O.


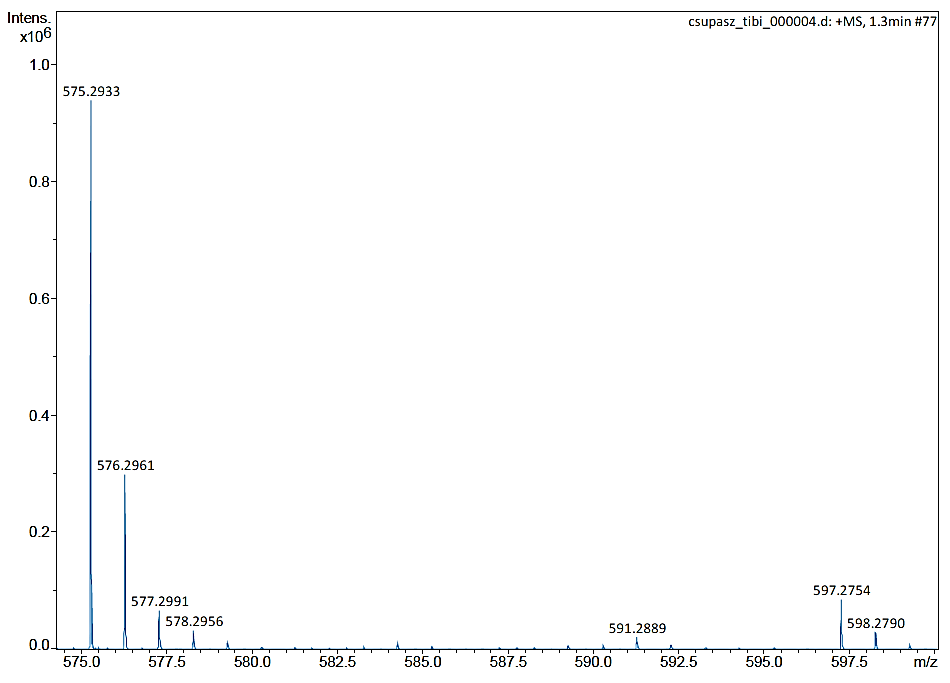


**Figure S7**. ESI HR-MS of the 3,9-PC2AM-NI ligand (m/z in positive mode).


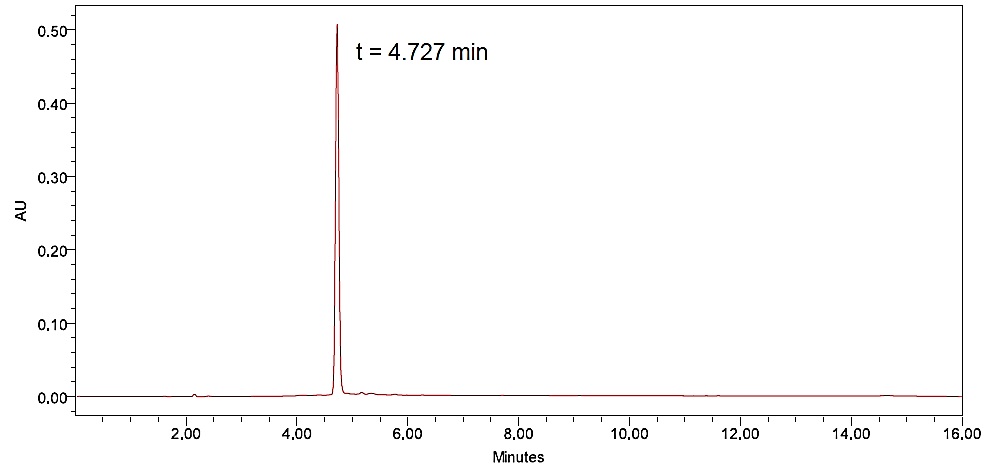


**Figure S8**. Analytical HPLC of the 3,9-PC2AM-NI ligand (*t*_R_ = 4.727 min, Eluent A: 5 mM TFA in water, Eluent B: MeCN; gradient: 95% A to 10% A over 12 minutes).


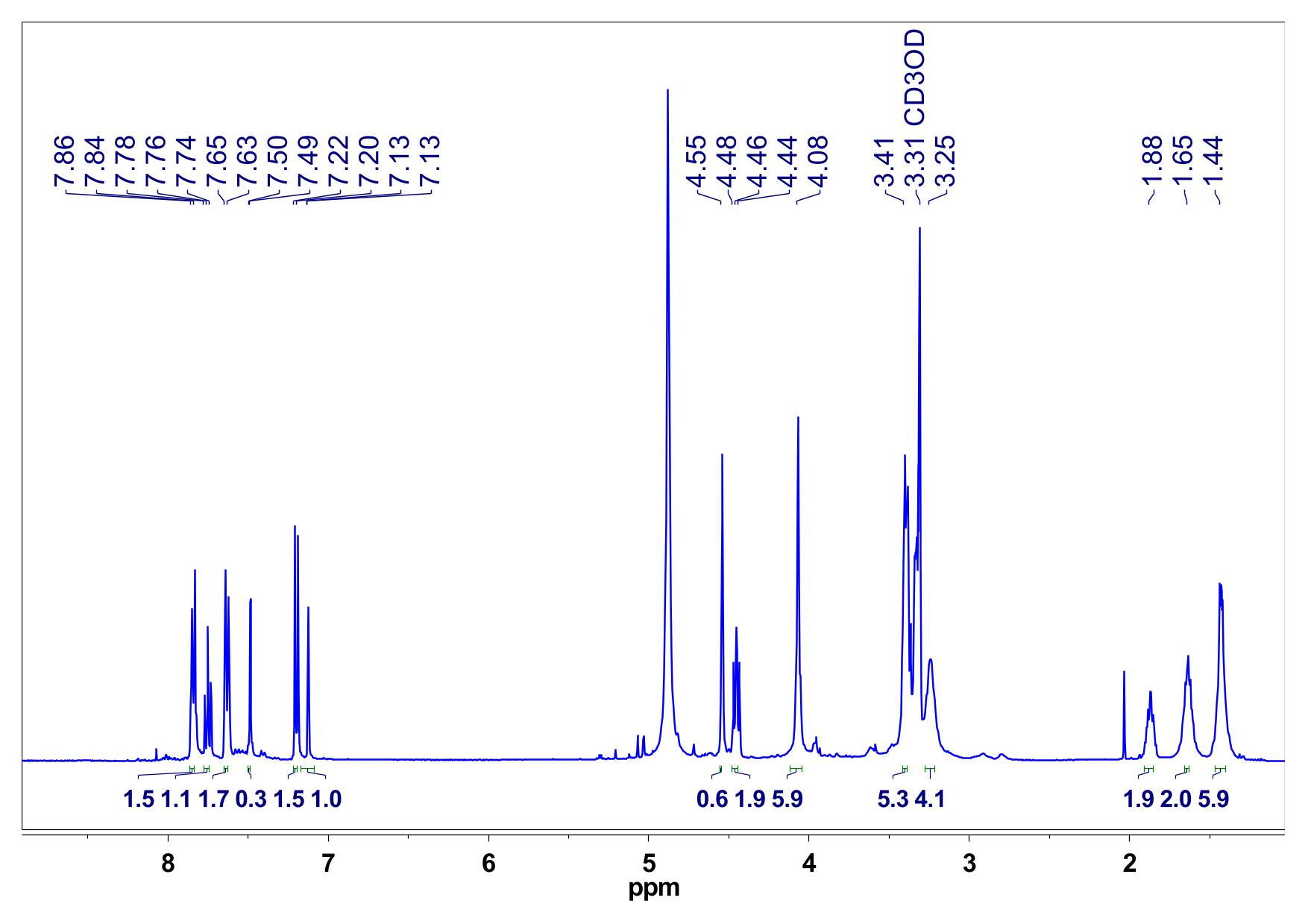


**Figure S9**. 360 MHz ^1^H NMR spectrum of the 3,9-PC2A-Ph-NI ligand in CD_3_OD.


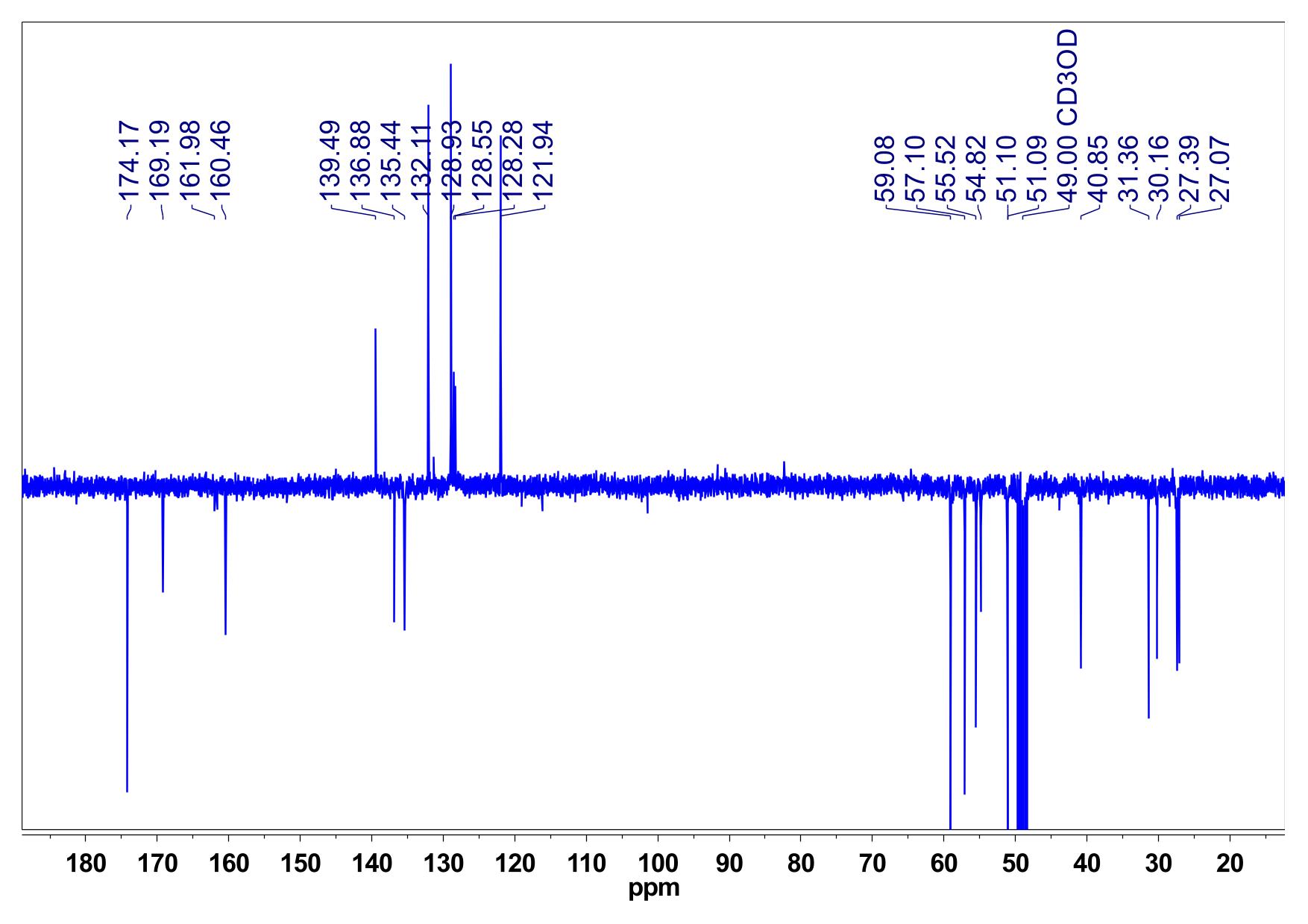


**Figure S10**. 90.55 MHz ^13^C NMR spectrum of the 3,9-PC2A-Ph-NI ligand in CD_3_OD.


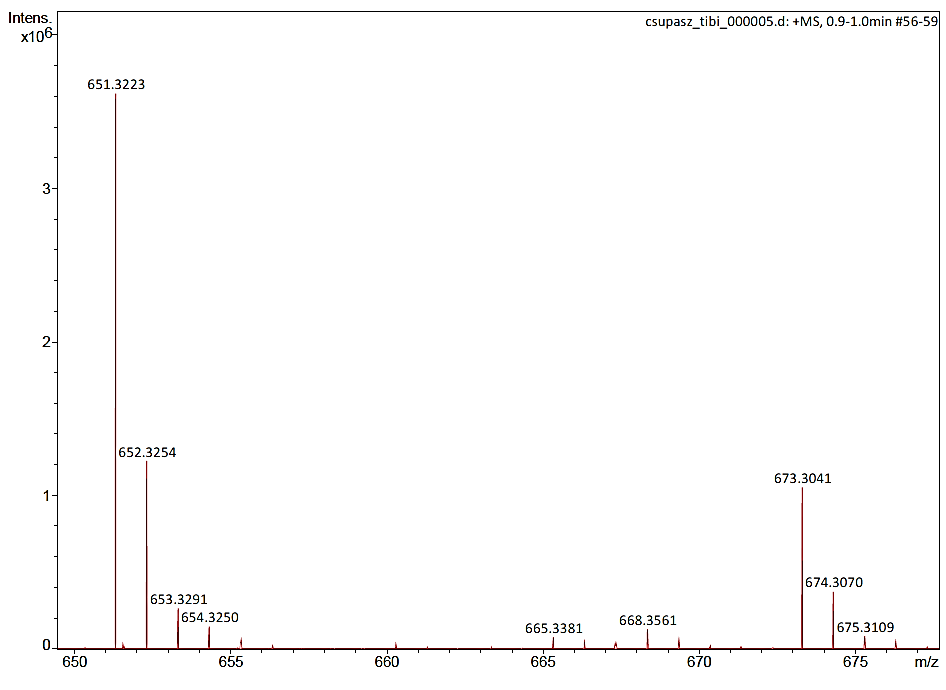


**Figure S11**. ESI HR-MS of the 3,9-PC2A-Ph-NI ligand (m/z in positive mode).


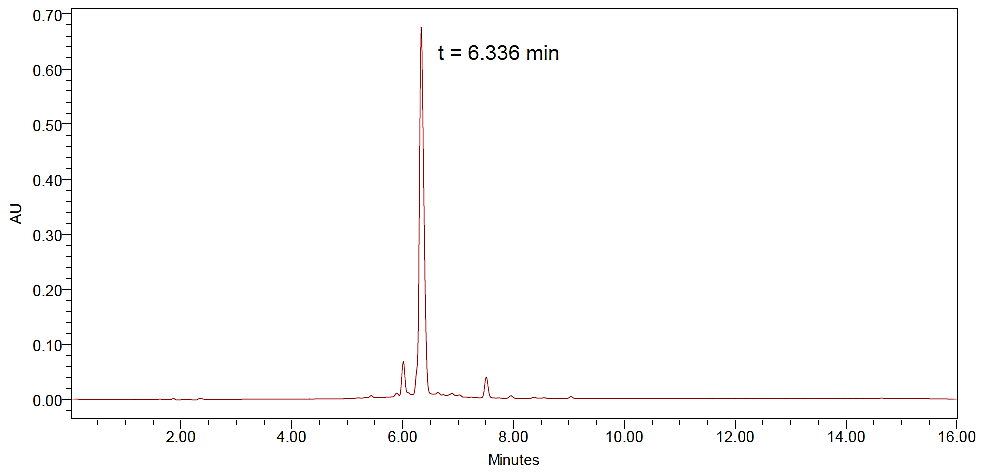


**Figure S12**. Analytical HPLC of the 3,9-PC2A-Ph-NI ligand (*t*_R_ = 6.336, Eluent A: 5 mM TFA in water, Eluent B: MeCN; gradient: 95% A to 10% A over 12 minutes).

**Figure S13**. Measured free Sc(III) concentration from ^45^Sc NMR (⚫ symbols) and calculated species distribution curves for the Sc(III) - (PCTA)^3-^ - H^+^ system as a function of-log[H^+^] values (c_Sc_= 3.88 mM, c_ligand_ = 3.95 mM, 1.0 M NaCl/HCl, 298 K).

**Figure S14**. Relative ligand affinity data (A_L/M_) calculated for Sc(III) complexes formed with PCTA, DO3AM-NI and PC2AM^nBu^ ligands as a function of pH as proposed by M. Meyer and co-workers (M. Meyer, N. Rollet, T.-H. Vu, S. Brandes and P. Gans, Acta Int. Symp. Metal Complexes, 2016, 6, 9).

**
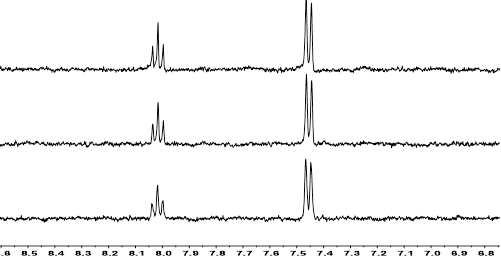
**

**Figure S15**. 400 MHz ^1^H-NMR spectra of [Sc(PC2AAM^nBu^)]^+^ (aromatic region) at different pH (3.1, 5.7 and 9.0) from top to bottom, respectively.

**Figure S16**. 400 MHz ^1^H-NMR spectra (aromatic region) of [Sc(PC2AAM^nBu^)]^+^ (c_ligand_ = 5 mM, upper trace) and equilibrium mixture of [Sc(PC2AAM^nBu^)]^+^ and [Sc(PC2AAM^nBu^)(F)] (c_ScL_= c_F_^-^ = 5mM, lower trace at pH = 5.50, *I* = 0.15 NaCl, T = 298K).

**Figure S17.** 97.20 MHz ^45^Sc NMR spectrum of the equilibrium mixture of [Sc(PC2AAM^nBu^)]^+^ and [Sc(PC2AAM^nBu^)(F)] (c_ScL_= c_F_^-^ = 5mM at pH = 5.50, *I* = 0.15 NaCl, T = 298K).

**Figure S18**. Kinetic profile of [Sc(PC2A1AMnBu)]^+^ formation monitored by ⁴⁵Sc NMR spectroscopy (⚫: free Sc(III), ⚫: complex) (c(Sc^3+^) = c(PC2AAM^nBu^) = 10 mM; pH = 1.28; *T* = 298 K).

**Figure S19.** Kinetic profile of [Sc(PCTA)] formation monitored by ⁴⁵Sc NMR spectroscopy (⚫: free Sc(III) ⚫: complex) (c(Sc^3+^) = c(PCTA) = 9.50 mM; pH = 1.28; *T* = 298 K).

**Figure S20.** UV spectra of 0.32 mM PC2A1AM^nBu^ ligand (blue) and that of 0.27 mM [Sc(PC2A1AM^nBu^)]^+^ complex (orange) in 1.0 M HClO_4_.

**Figure S21**. UV spectra of 0.30 mM PCTA ligand (blue) and that of 0.30 mM [Sc(PCTA)] complex (orange) in 2.6 M HClO_4_.

**Figure S22.** Dissociation kinetic profile of [Sc(PC2AAM^nBu^)]^+^ monitored by UV-vis spectroscopy in acidic medium (c_complex_ = 0.32 mM, λ = 280 nm, 2.6 M HClO_4_).

**Figure S23.** Dissociation kinetic profile of [Sc(PCTA)] monitored by UV-vis spectroscopy in acidic medium (c_complex_ = 0.30 mM, λ = 280 nm, 2.6 M HClO_4_).

**Figure S24.** 376.4 MHz ^19^F NMR spectrum recorded for the [Sc(PC2AAM^nBu^)(F)] (the signal, free fluoride (-120 ppm, ν_1/2_ = 4.5 Hz) and [Sc(PC2AAM^nBu^)(F)], -31 ppm, ν_1/2_ = 150 Hz) at pH = 5.20 (c_ScL_= c_F_^-^ = 5mM, *I* = 0.15 NaCl, T = 298K).

. -

**Figure S25.** Selective magnetization transfer (MT) experiment (intensity vs. delay time) curves for [Sc(PC2AAM^nBu^)(F)] (c_[Sc(PC2AAMnBu)(F)]_ = 5 mM, c_F_ = 10 mM, pH = 5.50, 298 K). The symbols show the measured intensities: red triangle [Sc(PC2AAM^nBu^)(F)] (site A); blue square, free F^-^ (site B), the curves represent the fitted data (*k*^obs^_BA_ = 1.2 s^-1^ (14% uncertainty), *k*^obs^_AB_ = 0.60 s^-1^ (14% uncertainty), *T*_1,A_= 0.211 s; *T*_1,B_ = 1.28 s (fixed)).

**
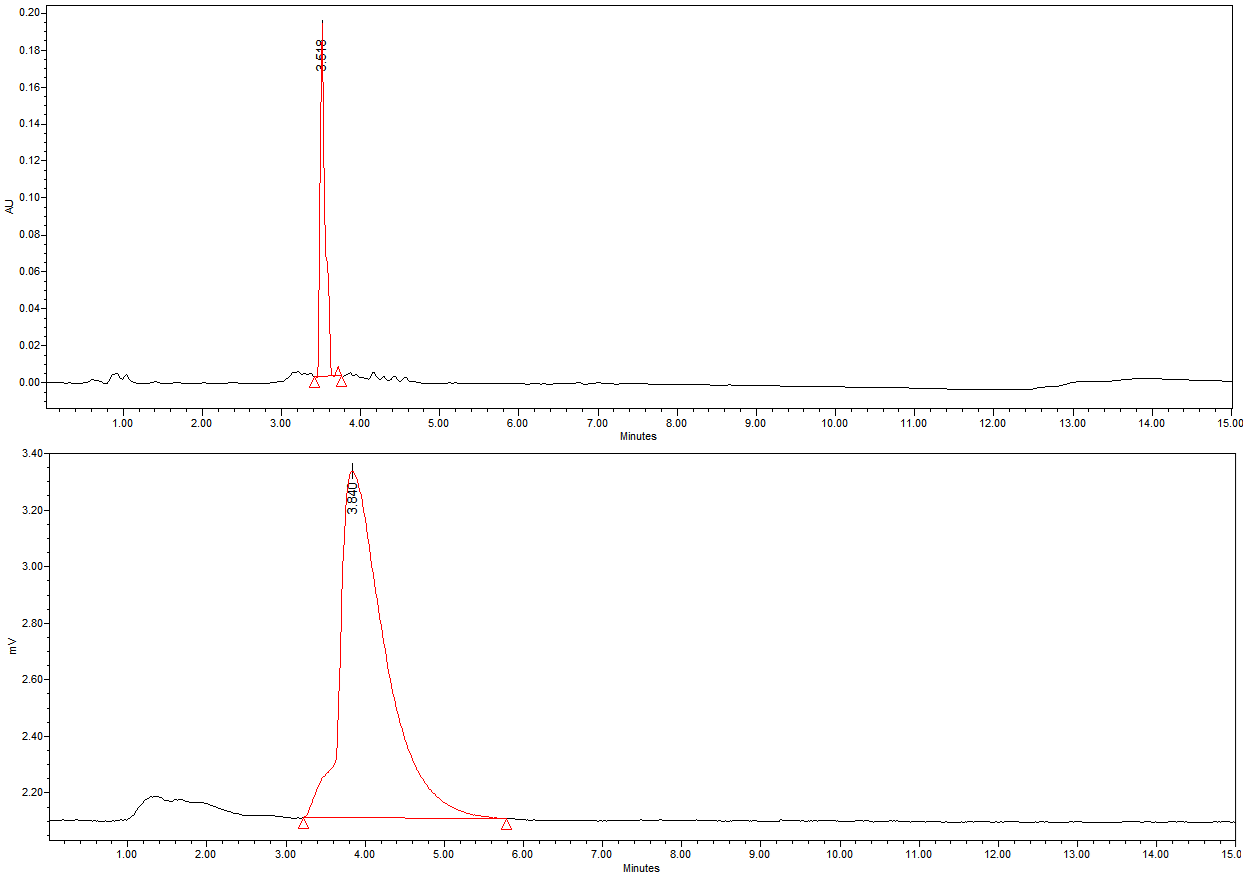
**

**Figure S26.** HPLC chromatograms of [^44^Sc][Sc(PC2AM-NI)]^+^with non-radioactive coinjection.


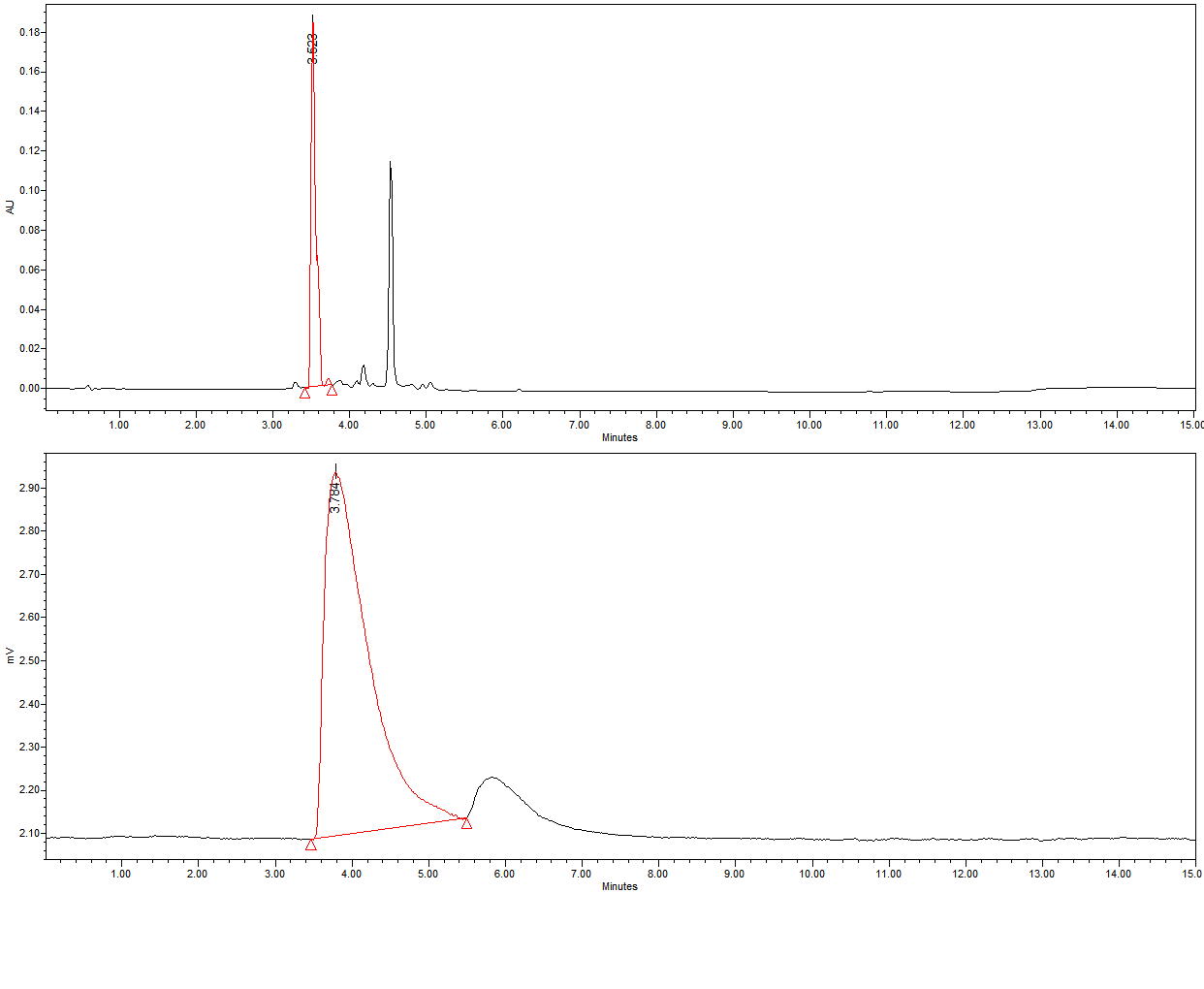


**Figure S27.** HPLC chromatograms [^44^Sc][Sc(PC2A-Ph-NI)]^+^ with non-radioactive coinjection.

***Formation of the ternary [^18^F][Sc(PC2AM^nBu^)(F)] complex***

Based on physicochemical studies demonstrating the ability of the [Sc(PC2AM^nBu^)]^+^ complex to bind fluoride, we decided to investigate the formation of the corresponding radiofluorinated ternary complex.

**[^18^F]fluoride production**:

[^18^F]fluoride was produced via the ^18^O(p,n)^18^F nuclear reaction using a GE PETtrace cyclotron at *Department of Nuclear Medicine, Institute of Medical Imaging, University of Debrecen*, Hungary. ^18^O-enriched water (>95 % [^18^O]H_2_O, Rotem Industries Ltd., Israel) was irradiated with 16 MeV protons (70 A). After irradiation the target was rinsed with deionized water, yielding approximately 1 GBq [^18^F]fluoride, which was isolated from the aqueous solution using SepPak QMA light anion-exchange cartridge. The trapped [^18^F]fluoride was washed with 1 mL water, and subsequently eluted with 1.000 mL of 0.4 M potassium bicarbonate.

**Radiochemical synthesis of the ternary [^18^F][Sc(PC2AM^nBu^)(F)] complex:**

A 50 µL of the this [^18^F]F stock solution was mixed with 50 µL of ammonium acetate buffer (1 M, pH = 4.5) followed by the addition of 20 µL of a ScCl_3_ stock solution (2 mM, 20 nmol). After incubation at room temperature for 10 minutes, 50 μL of PC2AMnBu stock solution (1 mg/mL, 100 nmol) was added. The mixture was then incubated at 110 °C for 30 minutes. The radiochemical conversion was 55.6%, as determined by radio-HPLC using a Luna C18 (3 μm, 150 × 4.6 mm) column with solvent A: water and solvent B: acetonitrile. The flow rate was 1.0 mL/min and the gradient was as follows: 0–1 min, 100% A; 1–10 min, linear gradient to 100% B; 10.1 min, return to 100% A. (Figure S28)


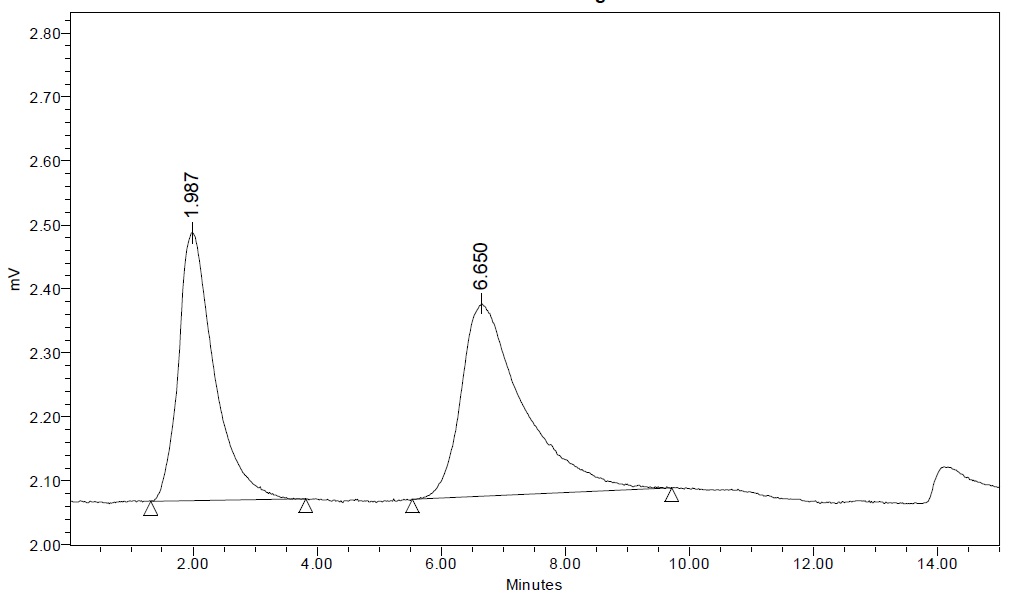


**Figure S28.** RadioHPLC chromatogram of crude reaction mixture of [^18^F][Sc(PC2AM^nBu^)(F)] complex (t_R_= 6.65 min: [^18^F][Sc(PC2AM^nBu^)(F)] complex and t_R_=1,987: **[**^18^F][Sc-F]^2+^).

This result provides a promising proof of concept experiment for the synthesis of radiofluorinated ternary complexes based on [Sc(PC2AM-NI)]^+^ and [Sc(PC2A-Ph-NI)]^+^ chelates.

[1] C.A.A. Kelderman, O.M. Glaser, J.N. Whetter, et al. Charting the coordinative landscape of the ^18^F–Sc/^44^Sc/^177^Lu triad with the tri-aza-cyclononane (TACN) scaffold. *Chem. Sci.*, **2024**, 15, 17927-17936.

**Table S1.** Summary of data used for the calculation of the stability constant, log *K*_[Sc(PC2AM_^nBu^_)(F)]_ by titration of 0.005M [Sc(PC2AM^nBu^)]^+^ parent complex with different amounts of F^-^ (0.005 - 0.01 M concentration range) followed by 376.4 MHz ^19^F NMR (I = 0.15 M NaCl, c _Sc(PC2AM_^nBu^) = 5 mM, pH=5.20, T= 298 K).

| Conc. of [Sc(PC2AM^nBu^)]^+^ (M) | Conc. of the F^-^ (M) | Integral ratio of the bound F^-^ | Integral ratio of the free F^-^ | *K*= [Sc(L)F]/[Sc(L)]*[F^-^] | log *K* |
| --- | --- | --- | --- | --- | --- |
| 0.0050 | 0.0050 | 1.00 | 1.08 | 356.65 | 2.55 |
| 0.0050 | 0.0060 | 1.00 | 0.99 | 508.89 | 2.71 |
| 0.0050 | 0.0070 | 1.00 | 1.40 | 342.86 | 2.54 |
| 0.0050 | 0.0080 | 1.00 | 1.96 | 222.09 | 2.35 |
| 0.0050 | 0.0100 | 1.00 | 2.80 | 150.79 | 2.18 |
